# The survival and function of IL-10-producing regulatory B cells are negatively controlled by SLAMF5

**DOI:** 10.1038/s41467-021-22230-z

**Published:** 2021-03-25

**Authors:** Lihi Radomir, Matthias P. Kramer, Michal Perpinial, Nofar Schottlender, Stav Rabani, Keren David, Anna Wiener, Hadas Lewinsky, Shirly Becker-Herman, Rina Aharoni, Ron Milo, Claudia Mauri, Idit Shachar

**Affiliations:** 1 Department of Immunology, The Weizmann Institute of Science, Rehovot, Israel; 2 Department of Neurology, Barzilai University Medical Center, Ashkelon, Israel; 3 Faculty of Health Sciences, Ben-Gurion University of the Negev, Beer-Sheva, Israel; 4 Centre for Rheumatology Research, Department of Medicine, University College London, London, UK

**Keywords:** Autoimmunity, Interleukins, Immunosuppression, B cells

## Abstract

B cells have essential functions in multiple sclerosis and in its mouse model, experimental autoimmune encephalomyelitis, both as drivers and suppressors of the disease. The suppressive effects are driven by a regulatory B cell (Breg) population that functions, primarily but not exclusively, via the production of IL-10. However, the mechanisms modulating IL-10-producing Breg abundance are poorly understood. Here we identify SLAMF5 for controlling IL-10^+^ Breg maintenance and function. In EAE, the deficiency of SLAMF5 in B cells causes accumulation of IL10^+^ Bregs in the central nervous system and periphery. Blocking SLAMF5 in vitro induces both human and mouse IL-10-producing Breg cells and increases their survival with a concomitant increase of a transcription factor, c-Maf. Finally, in vivo SLAMF5 blocking in EAE elevates IL-10^+^ Breg levels and ameliorates disease severity. Our results suggest that SLAMF5 is a negative moderator of IL-10^+^ Breg cells, and may serve as a therapeutic target in MS and other autoimmune diseases.

## Abstract (web summary)

Regulatory B (Breg) cells suppress excessive inflammation primary via the production of interleukin 10 (IL-10). Here the authors show that the function and homeostasis of mouse and human IL-10^+^ Breg cells are negatively regulated by the cell surface receptor, SLAMF5, to impact experimental autoimmunity, thereby hinting SLAMF5 as a potential target for immunotherapy.

## Introduction

Multiple sclerosis (MS) is an autoimmune disease of the central nervous system (CNS) leading to chronic neurological disabilities. It is characterized by extensive inflammation and demyelination, accompanied by axonal and neuronal damage. The disease is heterogeneous in its clinical manifestation and progression, as well as in its pathological mechanisms^1^.

MS has been considered a primarily T-cell-mediated disease in which T helper (Th)-1 and Th17 effector cells react against myelin components of the CNS, initiating a vicious inflammatory cascade in the CNS. In recent years, it was demonstrated that B cells are also essential for MS progression. B-cells activate and support the T-cell response through secretion of pro-inflammatory cytokines and were suggested to act as antigen-presenting cells (APC)^2^. In addition, autoantibodies against elements of the CNS are found in the cerebrospinal fluid (CSF) of MS patients^3,4^ and serve as a biomarker for the disease^5^. These immunoglobulins (Ig) were shown to localize within lesions and near damaged myelin and were demonstrated, ex vivo and in vitro, to cause axonal damage by inducing complement-mediated tissue injury^3,6^.

Essential data on MS have been obtained using the animal model, experimental autoimmune encephalomyelitis (EAE), induced by immunization of mice with myelin-derived antigens, such as myelin oligodendrocyte glycoprotein (MOG)^7,8^. MOG-induced EAE models are divided into B-cell-dependent disease, induced by the recombinant protein MOG_1-117_ (rMOG), and B-cell-independent disease, induced by the peptide MOG_35-55_^9^. In the rMOG model, B cells are activated by MOG and act as antibody-secreting cells and APCs. B-cell depletion in this model mitigates the disease and reduces effector T-cell activity^9^. B cells in the MOG_35-55_ model are not activated by this MOG form and are not required for disease development, and their depletion leads to clinical exacerbation. The pathogenesis of this model is mediated mainly by CD4^+^ effector T cells^9–11^, while Treg and Th2 responses may offer protection from the disease^12–14^.

In addition to the detrimental role of B cells in MS, B cells are also essential in disease mitigation^15^ and restriction of inflammation. This is achieved by regulatory B cells (Bregs), a subpopulation of B cells that is functionally characterized by its ability to suppress the immune response. Bregs were shown, both in humans and in mice, to maintain immune tolerance via diverse regulatory mechanisms, including the secretion of the anti-inflammatory cytokine, IL-10 IL-35, TGF-β, and by the expression of FasL, GITRL, PD-1, and CD73^16,17^. Mice lacking B cells, due to their genetic background or B-cell-depleting treatments^18–20^, could not recover from the disease, while the transfer of IL-10-producing B cells suppressed the inflammation and reduced disease severity^21,22^.

In humans and mice, Bregs comprise a group of B-cell subpopulations identified by specific markers. Even though several transcription factors (TFs) were found to be important for Bregs, none of these were found to be expressed by all the Breg populations or to act as a common identifying marker. Among their various functions, Bregs maintain the balance between Tregs and Th1/Th17 populations; in addition, they prevent differentiation of naive T cells into Th1 and Th17 while inducing Treg differentiation and cell expansion, which further suppresses pathological Th1 and Th17 responses^23,24^.

Cross-talk of B cells with their environment is crucial for their survival, activation, and differentiation. The signaling lymphocytic activating molecules (SLAM) family of immunomodulating receptors helps mediate the interaction of immune cells with their microenvironment^25^. This family, which consists of nine members, is differentially expressed mainly on immune cells. In most SLAM members, the extracellular segment is composed of two Ig-like domains, and they contain multiple immunoreceptor tyrosine-based switch motifs (ITSMs) on their cytoplasmic tail.

SLAMF5 (CD84), a member of the SLAM family, is a self-ligand receptor that forms homophilic dimers^26–28^. During cell-to-cell interactions, it acts both as an adhesion and signaling molecule^29^. Following its activation, SLAMF5 recruits adaptors of the SLAM adaptor protein (SAP) family which induces downstream signaling. SLAMF5 was previously reported to mediate the interactions between chronic lymphocytic leukemia (CLL) cells and their microenvironment^30^, leading to the support of CLL survival^31^ and to the suppression of T-cell activity via PD-L1^32^. Although SLAMF5 has an essential role in the regulation of the immune response, its direct role in autoimmunity was yet to be examined.

In this study, we investigate the role of SLAMF5 in EAE. We show that abrogating SLAMF5 interactions, by genetic deficiency or blocking antibody, alleviates disease in MOG-induced EAE mice while inducing the accumulation of IL-10-expressing Bregs. In vitro blocking of SLAMF5 expressed on B cells leads to a specific increase of Breg viability and induces expression of the IL-10-regulating transcription factor, c-MAF. These results support the role of the SLAMF5 receptor in modulating the accumulation of IL-10-producing Bregs during autoimmunity.

## Results

### SLAMF5 enhances disease severity in EAE

To examine the effect of SLAMF5 in EAE, we injected wild-type (WT) and SLAMF5-deficient (*slamf5*^−/−^) mice with MOG_35-55_ peptide in order to induce EAE. We monitored disease progression over 26 days using the EAE scoring system and changes in body weight.

**Fig. 1:**
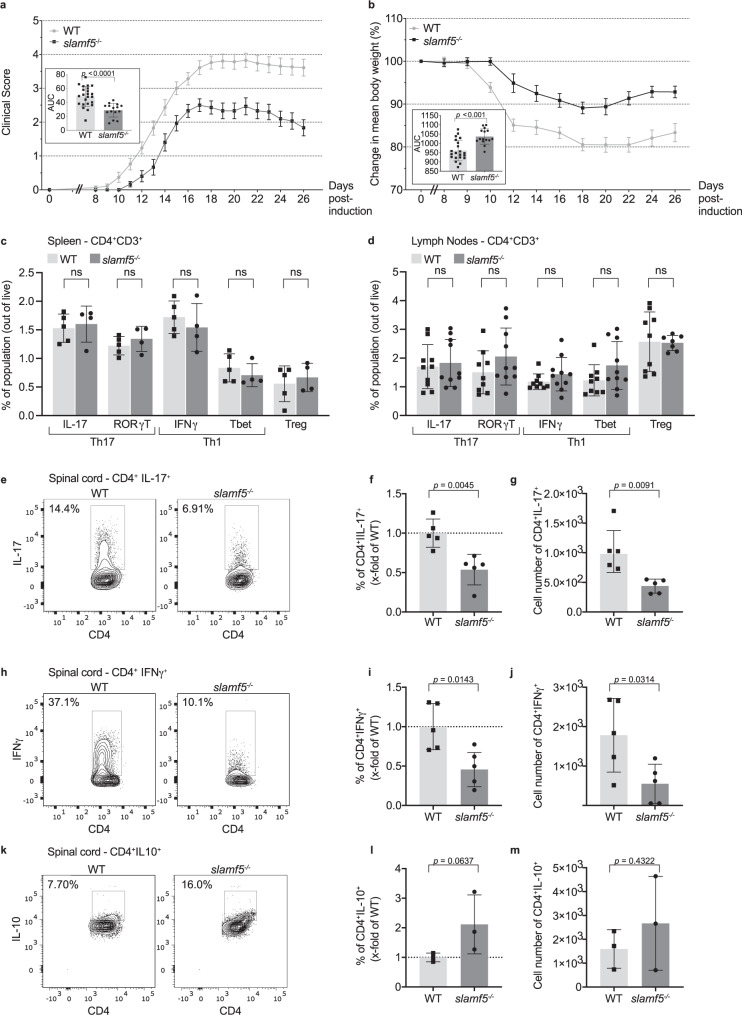
SLAMF5 deficiency mitigates EAE and increases Breg levels. EAE (MOG_35-55_) was induced in WT and *slamf5*^−/−^ mice. Mice were followed for 26 days. **a**) Daily mean clinical scoring of the disease (WT group *n* = 23; *slamf5*^−/−^ group *n* = 15, two representative independent experiments out of seven similar experiments). **b** Change in mean body weight shown as percent of initial body weight (WT group *n* = 21; *slamf5*^−/−^ group *n* = 15, two representative independent experiments out of seven similar experiments). **c**, **d** On day 15, spleens, lymph nodes (LNs), and spinal cords were collected, leukocytes were incubated overnight with MOG_35-55_ and analyzed by FACS for Th1 T cells (IL-17^+^ and RORγT^+^), Th17 T cells (IFNγ^+^ and T-bet^+^), and Treg (CD25^+^ FOXP3^+^) in (**c**) spleen (WT *n* = 5; *slamf5*^−/−^ *n* = 4, one experiment) (**d**) LNs, with gating for CD3^+^CD4^+^. (WT *n* = 9–10; *slamf5*^−/−^ *n* = 7–10, two independent experiments). **e**–**m** Four to five spinal cords were pooled together and analyzed for cytokine expression; showing representative histograms, percentage of secreting population, and cell count for: **e**–**g** IL-17^+^ (WT *n* = 5; *slamf5*^−/−^ *n* = 5, one experiment), **h**–**j** IFNγ^+^ T (WT *n* = 5; *slamf5*^−/−^ *n* = 4, one experiment), and **k**–**m** IL-10^+^ (WT *n* = 3; *slamf5*^−/−^ *n* = 3, one experiment) under the gate of CD45^+^CD11b^−^CD3^+^CD4^+^. Data expressed as mean ± s.e.m. (**a**, **b**) and mean ± s.d. (**a**, **b** insets, **c**, **d**, **f**, **g**, **i**, **j**, **l**, **m**). Mann–Whitney test (**a**, **b** insets). Unpaired Student’s *t* test with 95% confidence levels two-tailed (**c**, **d**, **f**, **g**, **i**, **j**, **m**), one-tailed (**l**). (***P < 0.001, ****P < 0.0001). Gating strategy for **c** and **d** is shown in Supplementary Fig. 1a(I.-IV.), gating strategy for **e**–**m** is shown in Supplementary Fig. 1g.

The clinical impairment of WT mice typically appeared 10 days after disease induction, increasing in severity and reaching a mean clinical score of 3.76 ± 0.2 by day 18, at which point disease incidence was 100%. In contrast, *slamf5*^−/−^ mice exhibited delayed disease onset and milder motor dysfunction with a maximal mean score of 2.1 ± 0.25 at day 18, and incidence of only 78% (Fig. 1a and Supplementary Fig. 1a). Differences between WT and *slamf5*^−/−^ mice were already evident from day 12 and persisted until the end of the experiment. Furthermore, the difference was found significant according to the area under the curve graphing disease severity (AUC, inset in Fig. 1a). The amelioration of disease was accompanied by reduced weight loss in the *slamf5*^−/−^ compared to the WT group (Fig. 1b, AUC in inset), together indicating that *slamf5*^−/−^ mice are partially protected from EAE.

Since Th1 and Th17 effector cells promote EAE progression, and recovery is promoted by Treg cells, we analyzed these populations. During the active phase of the disease (day 15), we found no differences in the Th1 (Tbet and effector cytokine IFNγ) or the Th17 population (RORγT and effector cytokine IL-17) in the spleen or lymph nodes (Fig. 1c, d). Furthermore, no differences were found in the Treg (FOXP3^+^CD25^+^, Fig. 1c, d), total CD4 (Supplementary Fig. 1c), or Th2 (Gata3 and cytokine IL-4, Supplementary Fig. 1d, e) populations. Examination of the cytokine TNFα, which supports inflammation in EAE^33^, also showed no differences between WT and *slamf5*^−/−^ (Supplementary Fig. 1f).

The main site of inflammation in the MOG-induced EAE model is the spinal cord (SC)^34^. Due to the low number of T cells in the SC, we pooled the SCs of four to five mice to perform staining for effector cytokines. Interestingly, the percent and numbers of CD4^+^IL-17^+^ (Fig. 1e–g) and CD4^+^IFNγ^+^ T cells (Fig. 1h–j) were reduced in *slamf5*^−/−^ mice, accompanied by an increase of CD4^+^IL-10^+^ T cells (Fig. 1k–m).

This downregulation of effector T cells in the SC may be due to a disruption in antigen presentation by microglia, monocytes (perivascular macrophages and dendritic cells), and/or B cells residing in the SC^35–37^. However, no differences were found in the levels of MHC class II expression on these populations (Supplementary Fig. 1h, i), indicating that antigen presentation is intact, and the effect of SLAM5 deficiency must be due to another mechanism.

### SLAMF5 negatively controls IL-10^+^ Breg accumulation

**Fig. 2:**
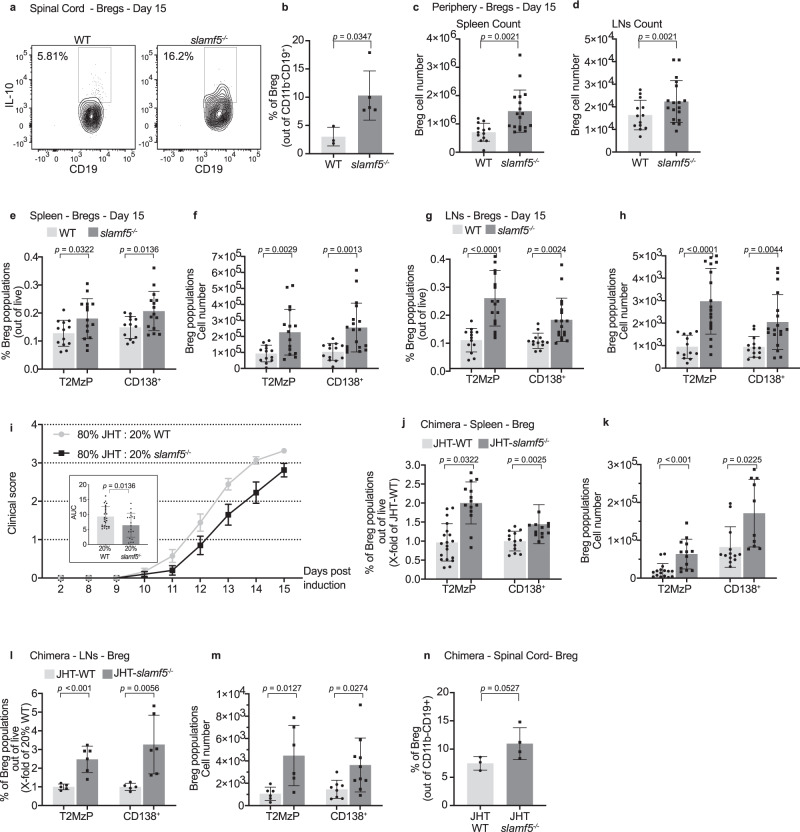
SLAMF5 deficiency regulates Breg levels. EAE (MOG_35-55_) was induced in WT and *slamf5*^−/−^ mice. **a**, **b** On day 15, the spinal cords were analyzed the for Breg population according to the following markers: CD45^+^CD11b^−^CD19^+^CDIL-10^+^; **a** representative dot plot, and **b** bar graph showing the mean population percentages. Each dot represents a pool of four to five mice with similar disease scores, (WT *n* = 3; *slamf5*^−/−^ *n* = 5, two independent experiments). **c**–**h** On day 15, spleens and lymph nodes (LNs) were analyzed. Total Breg (CD19^+^IL-10^+^) cell numbers in (**c**) the spleen and (**d**) LNs (WT *n* = 13; *slamf5*^−/−^ *n* = 18, two independent experiments), subpopulations of T2-MzP Breg (CD19^+^IL-10^+^CD24^+^CD21^+^CD23^+^) and CD138^+^ Bregs (CD19^+^IL-10^+^CD138^+^) were analyzed for percentages and cell number in (**e**, **f**) the spleen (WT *n* = 13; *slamf5*^−/−^ *n* = 15, two independent experiments) and (**g**, **h**) LNs (WT *n* = 13; *slamf5*^−/−^ *n* = 18, two independent experiments) (**i**–**n)** CD45.1 WT mice were lethally irradiated and injected with BM consisting of 80% JHT BM and 20% WT-CD45.2 or *slamf5*^−/−^ BM. Following BM reconstitution, EAE was induced, and mice were followed for 15 days. **i** Mean clinical scoring of the disease. Inset depicts the area under the curve for days 0–15 (20% WT *n* = 24; 20% *slamf5*^−/−^ *n* = 20, three independent experiments). On day 15, Breg populations were analyzed in the spleen by FACS for (**j**) percentages (20% WT T2MzP *n* = 19; CD138^+^ *n* = 13; 20% *slamf5*^−/−^ T2MzP *n* = 14; CD138^+^ *n* = 13, three independent experiments) and (**k**) cell number (20% WT T2MzP *n* = 16; CD138^+^ *n* = 13; 20% *slamf5*^−/−^ T2MzP *n* = 13; CD138^+^ *n* = 10, three independent experiments) . LN Bregs were analyzed for their (**l**) percentage (each dot represents four to five mice with a similar score, 20% WT *n* = 6; 20% *slamf5*^−/−^ *n* = 5, three independent experiments), and (**m**) cell count (20% WT T2MzP *n* = 6; CD138^+^ *n* = 8; 20% *slamf5*^−/−^ T2MzP *n* = 6; CD138^+^ *n* = 10, three independent experiments). **n** Spinal cord Bregs (each dot represents four to five mice with a similar score, 20% WT *n* = 3; 20% *slamf5*^−/−^ *n* = 4, two independent experiments) were analyzed. Data expressed as mean ± s.d. (**b**–**h**, **i** inset, **n**) and mean ± s.e.m. (**i**). Unpaired Student’s *t* test with 95% confidence levels two-tailed (**b**–**h**, **j**–**m**) or one-tailed (**n**), Mann–Whitney test (**i** insets). Gating strategy for **a**, **b**, **n** is shown in Supplementary Fig. 2a, gating strategy for **a**–**d**, **e**–**h**, **j**, **k** is shown in Supplementary Fig. 2b.

To further investigate the downregulation of effector T cells in the SC, we analyzed the population of B cells that exert regulatory function, suppress the CD4^+^ effector response, and produce IL-10 (IL-10^+^ regulatory B cells; IL-10^+^ Bregs or Bregs)^38^. Due to the low number of B cells in the SC, the SCs of four to five mice with matching scores were combined to perform IL-10 staining. To our surprise, we found a significant increase in Breg percentage in the SC of the *slamf5*^−/−^ mice (Fig. 2a, b).

To determine whether the Breg population is affected only in the SC, we next analyzed Bregs (CD19^+^IL-10^+^) in the lymphoid organs. We observed a significant increase in the numbers of Bregs on day 15, both in the spleen and LN (Fig. 2c, d). This increase was limited to the IL-10-expressing populations and was not detected in the IL-10^neg^ total B cells or in the B subpopulation; the mature, marginal zone (Mz), transitional 2 (T2), and transitional 1 (T1) (Supplementary Fig. 2c).

We next analyzed two of the major B-cell subsets subpopulations that have been previously shown to contain the majority of IL-10-producing B cells and were shown to play a role in the regulation of autoimmune diseases^20,39^; T2MzP Bregs (IL-10^+^CD24^+^CD21^hi^CD23^+^) and CD138^+^ Bregs (IL-10^+^CD138^+^). The percentages and absolute numbers of both IL-10-positive subpopulations were significantly elevated in *slamf5*^−/−^ mice compared to WT on day 15, both in the spleen (Fig. 2e, f) and in the LNs (Fig. 2g, h). At the start of the recovery phase, on day 26, an increase in the IL-10-producing Breg populations was detected in the spleen (Supplementary Fig. 2d, e), but not in the LNs of *slamf5*^−/−^ mice (Supplementary Fig. 2f, g). The elevation in Breg numbers was specific to the disease process since no change in Breg populations was observed in the spleens of WT and *slamf5*^−/−^ that were treated with adjuvant (CFA) alone (Supplementary Fig. 2h, i).

T2MzP B cells, which contain a subpopulation with the potential to become IL-10-producing cells, are mostly located in the spleen; however, T2MzP Bregs (CD23^high^CD21^high^IL-10^+^) were detected in the LNs of EAE mice (Supplementary Fig. 3a) and were previously reported in the draining LNs of melanoma tumors^40^. CD138^+^ B cells can be divided into plasmablasts (PB, CD138^+^CD44^hi^)^20^ and plasma cells (PC, CD138^+^CD44^low^)^41^. Both subpopulations were shown to consist of IL-10-secreting cells and to have an important role in EAE. Therefore, we followed the regulation of these IL-10-expressing subpopulations by SLAMF5 deficiency. As seen in Supplementary Fig. 3b–d, both populations were increased in the spleen and LNs of *slamf5*^−/−^ mice. Therefore, in the rest of the study, we continued analyzing the total IL-10^+^CD138^+^ population.

SLAMF5 deficiency can increase Breg numbers through either direct intrinsic signaling in the Bregs themselves or as a consequence of a change in SLAMF5 signaling in their microenvironment. In order to further unravel the mechanism of SLAMF5, we generated chimeric mice in which SLAMF5 deficiency is mostly restricted to the B cells, using bone marrow (BM) of JHT mice. These mice lack the J_H_ gene, leading to disruption in B-cell development, and resulting in a lack of early and mature B-cell populations^42^. Recipient WT-CD45.1 mice were irradiated and transplanted with a BM mixture of 80% JHT BM:20% *slamf5*^−/−^ or WT BM (Supplementary Fig. 4a). Chimerism and restriction of SLAMF5 deficiency mostly to the B-cell populations was confirmed (Supplementary Fig. 4b–d).

EAE was then induced in the JHT-chimeric mice and the disease course followed for 15 days. The clinical manifestation in the mice reconstituted with 20% WT cells, typically appeared 10 days after disease induction, increasing in severity and reaching a mean clinical score of 3.3 ± 0.07 by day 15, with disease incidence of 100%. The mice reconstituted with 20% *slamf5*^−/−^ cells manifested the disease on day 10, as well, but with a milder motor dysfunction with a reduced maximal mean score of 2.7 ± 0.21 by day 15, with 95% of the mice exhibiting the disease (Fig. 2i and Supplementary Fig. 4e). A significant overall difference was evident in the AUC (inset in Fig. 2i). The disease induction in the JHT:WT chimeras was similar to the development of the disease in wild-type mice (Fig. 1a) and to the disease observed when WT BM cells were transplanted into a WT recipient (Supplementary Fig. 4f).

Similar to the fully *slamf5*^−/−^ mice, we found a significant increase of T2MzP and CD138^+^ Bregs in the spleen (Fig. 2j, k). The LNs of the chimeric mice were small to undetectable, and therefore the LNs of four to five mice with matching scores were combined for analysis. An elevation was seen in T2MzP and CD138^+^ Bregs percentages and numbers in these chimeric mice (Fig. 2l, m). Furthermore, the Breg was significantly elevated in the SC of the 20% *slamf5*^−/−^ mice (Fig. 2n) recapitulating the results of the full SLAMF5-deficient mice. These results suggest that SLAMF5 is an intrinsic negative regulator of Breg abundance in the spinal cord and lymphoid tissues during the active phase of the disease.

### SLAMF6 does not play a role in Breg accumulation during EAE

Some of the roles of SLAMF5 overlap with those of other members of the SLAM family. SLAMF6 (Ly108 in mice, NTB-A in humans) regulates naive B-cells maintenance^43^ and the production of autoantibodies^44^. We, therefore, wished to determine whether regulation of Breg accumulation is mediated exclusively by SLAMF5 or whether an additional member of the family, SLAMF6 may have a similar function. SLAMF6 deficiency partially protected mice from EAE, but these mice showed a higher disease incidence of 95% (Supplementary Fig. 5a–c) compared to the *slamf5*^−/−^. However, lower levels of Bregs were detected in the SC of *slamf6*^−/−^ compared to the WT (Supplementary Fig. 5d), and no differences in the levels and numbers of total Bregs in the spleen (Supplementary Fig. 5e, f) and LNs (Supplementary Fig. 5g, h) were observed. Effector T-cell markers were lower in the LNs of *slamf6*^−/−^ mice compared to WT but were similar in the spleen (Supplementary Fig. 5i, j), suggesting changes in T-cell function in these mice. Further examination of the role of SLAMF6 in B cells during EAE was performed using JHT-*slamf6*^−/−^ chimeric mice, where SLAMF6 deficiency was mostly restricted to B cells. Disease progression showed no difference between JHT-WT and JHT-*slamf6*^−/−^ mice (Supplementary Fig. 4k). Similar to the full *slamf6*^−/−^ mice, no change in Breg levels and numbers was seen in the JHT-*slamf6*^−/−^ mice compared to JHT-WT (Supplementary Fig. 5l, m). These results suggest that SLAMF6 does not regulate the Breg populations in EAE.

### SLAMF5 is highly expressed on Bregs

**Fig. 3:**
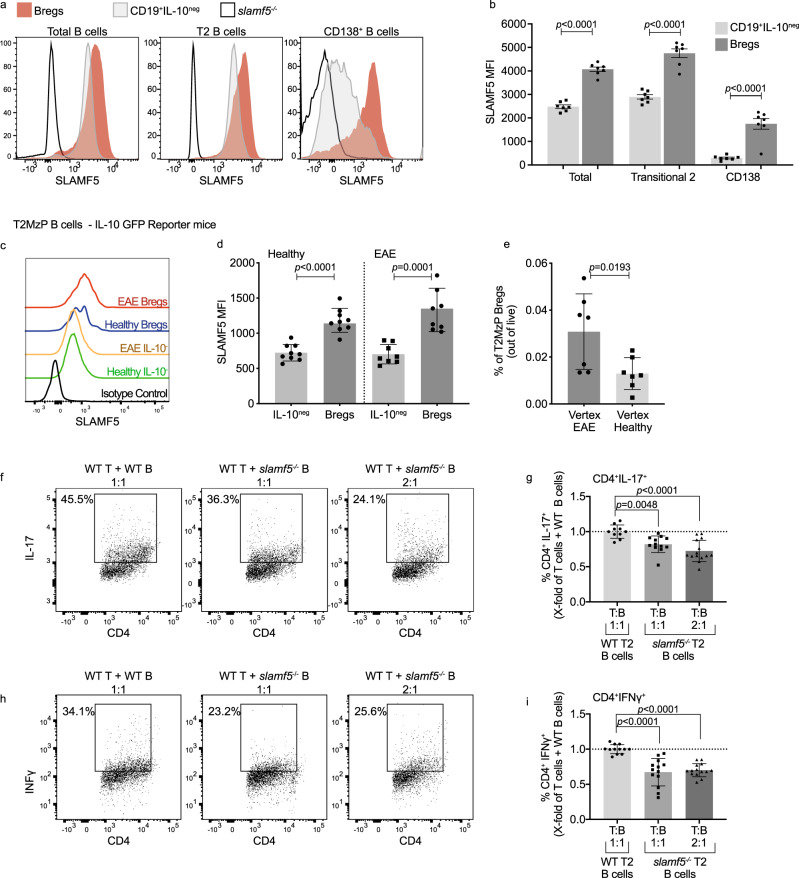
SLAMF5 is highly expressed on Bregs and regulates their functionality. **a**, **b** Analysis of SLAMF5 expression on splenic regulatory B compared to non-Bregs (B cells IL-10^neg^) of WT EAE (MOG_35-55_)-induced mice. **a** Representative histograms, and **b** bar charts showing the SLAMF5 MFI on B-cell subpopulations according to the following markers: total B cells (CD19^+^), T2 B cells (CD19^+^CD24^+^CD21^+^CD23^+^), and CD138^+^ B cells (CD19^+^CD138^+^). Populations are divided into IL-10^+^ (Bregs) and IL-10^neg^ (non-Bregs) (non-Bregs *n* = 7; Bregs *n* = 7, two independent experiments). **c**–**e** Spleens of Vert-x mice, healthy or EAE-induced, were harvested and analyzed for SLAMF5 expression on IL-10^+^ or IL-10^neg^ T2MzP cells. **c** Representative histogram and **d** bar graph showing SLAMF5 MFI. (EAE: Bregs *n* = 8; non-Bregs *n* = 8; healthy: Bregs *n* = 9 non-Bregs *n* = 9, two independent experiments). **e** Spleens were analyzed for percentages of T2MzP Bregs (IL-10^+^) (healthy *n* = 7; EAE *n* = 7, two independent experiments). **f**–**i** Sorted CD4^+^CD25^-^ splenic T cells of WT EAE-induced mice were co-cultured 1:1 or 2:1 for 72 h in the presence of anti-CD3 and anti-CD28 Abs with WT or *slamf5*^−/−^ sorted splenic transitional 2 B cells (CD19^+^CD23^+^CD21^+^). Cells were analyzed for (**f**–**g**) IL-17 expression, shown as (**f**) representative dot plots, and (**e**) bar graph (WT T:WT B 1:1 *n* = 10; WT T:*slamf5*^−/−^ B 1:1 *n* = 12; WT T:*slamf5*^−/−^ B 2:1 *n* = 14, five independent experiments) and (**h**, **i**) IFNγ expression, shown as (**h**) representative dot plots, and (**i**) bar graph. (WT T:WT B 1:1 *n* = 12; WT T:*slamf5*^−/−^ B 1:1 *n* = 13; WT T:*slamf5*^−/−^ B 2:1 *n* = 14, five independent experiments). Data expressed as mean ± s.e.m. (**b**) and mean ± s.d. (**d**, **e**, **g**, **i**). Unpaired Student’s *t* test with 95% confidence levels two-tailed (**b**, **d**, **e**) or ordinary one-way ANOVA with Dunnett multiple comparison tests (**g**, **i**). Gating strategy for **a** and **b** is shown in Supplementary Fig. 2b, gating strategy for **c** and **d** is shown in Supplementary Fig. 6d, gating strategy for **h** and **i** is shown in Supplementary Fig. 1b (I., III.).

To follow the role of SLAMF5 in Bregs, we analyzed SLAMF5 expression on different splenic B-cell populations derived from EAE-induced mice. The different subpopulations of B cells were evaluated for SLAMF5 expression on either IL-10-positive (Bregs) or IL-10-negative (non-Breg) populations. Increased levels of SLAMF5 were detected on Bregs compared to its expression on their non-Breg B-cells counterparts, with the highest levels on the T2MzP Bregs (Fig. 3a, b). High levels of SLAMF5 were also detected on Bregs derived from WT mice injected only with CFA relative to non-Breg populations (Supplementary Fig. 6a).

IL-10 staining requires in vitro activation, in which cells are stimulated with the TLR4 agonist lipopolysaccharide (LPS). To verify that the high SLAMF5 expression levels on Bregs did not result from the in vitro stimulation, SLAMF5 levels were analyzed on Vert-x mice which express a GFP reporter on the IL-10 gene, which eliminates the need for TLR4 activation^45^. T2MzP Bregs expressed higher levels of SLAMF5 compared to their IL-10^neg^ counterparts in both EAE-induced and healthy Vert-x mice (Fig. 3c, d). In addition, although Breg frequencies were higher in mice induced for EAE (Fig. 3e), Bregs from EAE-induced or healthy Vert-x mice expressed similar levels of SLAMF5. This higher SLAM5 expression was also observed on total Bregs compared to total non-Bregs (Supplementary Fig. 6b, d) and CD138 Bregs compared to non-Breg CD138 cells (Supplementary Fig. 6c, d), suggesting that Bregs highly express SLAMF5 regardless of disease or TLR activation.

To further explore the effect of TLR activation on SLAMF5 expression by Breg, its expression was analyzed on in vitro generated Bregs. B cells from naïve mice were incubated for 24 or 72 h in the presence of LPS. An increase in the Breg population was observed throughout the experiment, while IL-10^neg^ B-cell levels were reduced (Supplementary Fig. 6e). These Bregs expressed higher levels of SLAMF5 compared to non-Bregs cultured under the same conditions (Supplementary Fig. 6f). However, SLAMF5 expression levels did not change during the incubation time (Supplementary Fig. 6f) on either population, indicating that LPS does not affect SLAMF5 expression levels, further suggesting that SLAMF5 is an intrinsic feature of regulatory B cells.

### SLAMF5 negatively regulates Breg function

Next, we wished to determine whether SLAMF5 regulates Breg functionality. Analysis of IL-10 expression in WT and *slamf5*^−/−^ Bregs revealed similar levels of this cytokine in both populations (Supplementary Fig. 6g, h). To investigate Breg functionality, a Breg suppression assay was performed. WT or *slamf5*^−/−^ transitional 2 (T2) B cells, containing the T2MzP Breg population, were cultured with WT CD25^−^CD4^+^ T cells at 1:1 ratio. As previously shown^46^, the suppression of effector T-cell function is partially mediated by IL-10 secretion (Supplementary Fig. 7a). The abundance of Bregs in the *slamf5*^−/−^ group was twofold higher than Bregs in the WT T2 B cells. Therefore, to normalize the number of Bregs in the two groups, WT or *slamf5*^−/−^ T2 cells were cultured with WT CD25^-^CD4^+^ T cells at a 2:1 ratio. After 72 h, cells were analyzed for the expression of the cytokines IL-17 and IFNγ. T cells cultured with *slamf5*^−/−^ B cells showed a significantly lower level of the cytokines IL-17 (Fig. 3f, g and Supplementary Fig. 7b) and IFNγ (Fig. 3h, i and Supplementary Fig. 7c), suggesting stronger suppression by *slamf5*^−/−^ T2 B cells of T-cell differentiation relative to wild-type.

The reduction of T-cell cytokine expression in the presence of *slamf5*^−/−^ T2 B cells may result from insufficient T- and B-cell interactions through SLAMF5 rather than intrinsic changes in Breg functionality. However, while deficiency of SLAMF5 on B cells increased T-cell suppression, SLAMF5 deficiency on the T cells themselves had no effect on their suppression (Supplementary Fig. 7d, e). In addition, analysis of T–B cell conjugate formation in the suppression assay by analysis of CD19–CD4 doublets^43^, showed similar levels of the doublets when SLAMF5 deficiency was on the B cells or on the T cells (Supplementary Fig. 7f–h). Thus, SLAMF5 deficiency did not impair the interactions between B cells and T cells and therefore the elevated suppression detected in the presence of SLAMF5-deficient B cells resulted from a factor intrinsic to these B cells.

### Breg survival is regulated by SLAMF5

**Fig. 4:**
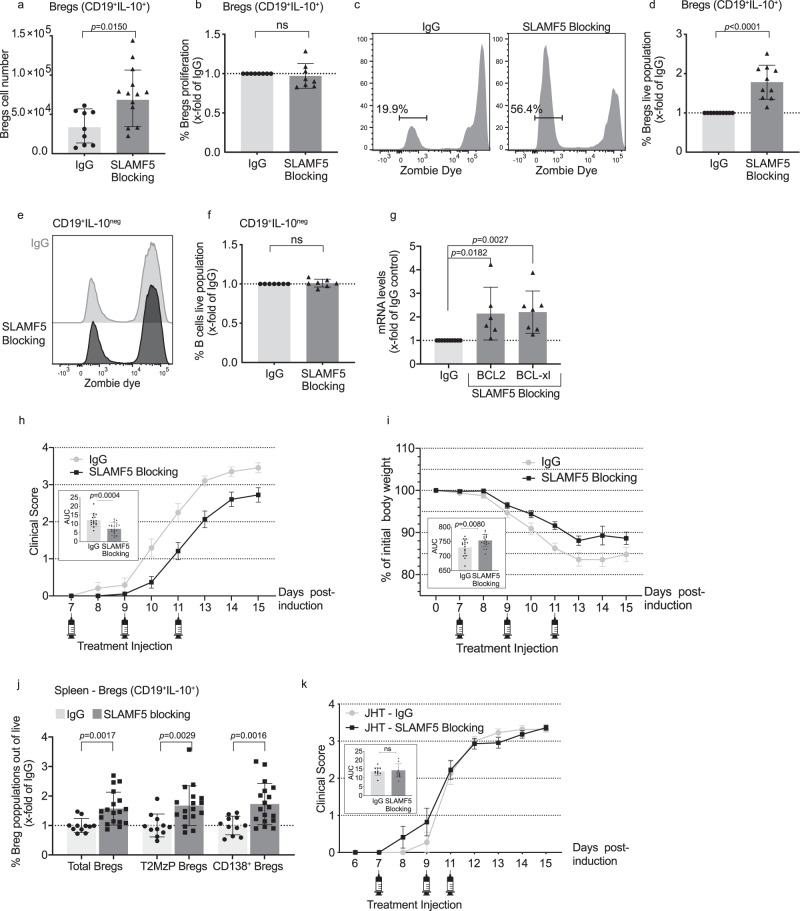
SLAMF5 blocking increases Breg survival in vitro and increases Breg numbers in vivo. **a**–**f** Splenic B cells derived from EAE-induced WT mice were cultured and treated with LPS and either SLAMF5-blocking or IgG-control antibodies for 48 h, with PMA, ionomycin, and monensin for the final 5 h. Cells were collected, and the survival of Bregs and non-Breg B cells (CD19^−^IL-10^neg^) was analyzed using Zombie dye by flow cytometry; **a** Breg cell numbers (IgG *n* = 9; SLAMF5-blocking *n* = 15, five independent experiments), **b** Breg proliferation (IgG *n* = 8; SLAMF5-blocking *n* = 8, three independent experiments), **c**, **d** Breg survival showing (**c**) representative histograms of Zombie dye staining (live dead staining), and **d** percentage of live (Zombie^neg^) Breg shown as *x*-fold of IgG (IgG *n* = 10; SLAMF5-blocking *n* = 10, four independent experiments). **e**, **f** Non-Breg B-cell survival: **e** representative histograms of Zombie dye staining and (**f**) graph summarizing the percentage of live (Zombie^neg^) B cells (IgG *n* = 7; SLAMF5-blocking *n* = 7, three independent experiments). **g** B-cell pool of two to three EAE-induced Vert-x mice was sorted for Bregs (CD19^+^GFP^+^), cultured, and treated with SLAMF5-blocking Ab or IgG-control for 24 h. Cells were analyzed for mRNA levels of Bcl-2 and Bcl-xl by qPCR analysis. Data are shown as *x*-fold of treatment compared to IgG-control. Each dot represents a combination of two to three score-matched mice (Bcl-2: IgG *n* = 6; SLAMF5-blocking *n* = 6, two independent experiments, Bcl-xl: IgG *n* = 7; SLAMF5-blocking *n* = 7, two independent experiments). **h**–**j** EAE was induced in WT mice. On days 7, 9, and 11 the mice were injected i.v. with 30 µg SLAMF5-blocking Ab or IgG. **h** Daily mean clinical scoring of the disease (SLAMF5-blocking *n* = 18; IgG *n* = 17; three independent experiments). **i** Mean weight loss during the disease course shown as percent of initial body weight. Inset depicts the area under the curve for days 0–15. (SLAMF5-blocking *n* = 19; IgG *n* = 17; three independent experiments). **j** On day 15, spleens were collected and analyzed for Breg populations; total Bregs (CD19^+^IL-10^+^), T2MzP (CD19^+^IL-10^+^CD24^+^CD23^+^CD21^+^) and CD138^+^ Bregs (CD19^+^IL-10^+^CD138^+^). (SLAMF5-blocking: total *n* = 18; T2MzP *n* = 17; CD138 *n* = 18; IgG: total *n* = 11; T2MzP *n* = 11; CD138 *n* = 11, three independent experiments). **k** EAE was induced in JHT mice. On days 7, 9, and 11 the mice were injected i.v. with 30 µg SLAMF5-blocking Ab or IgG. Daily mean clinical scoring of the disease. Insets depict the area under the curve for days 0–15. (SLAMF5-blocking *n* = 11; IgG *n* = 11; two independent experiments. Data expressed as mean ± s.d. (**a**–**d**, **f**, **g**, **h**, **i** insets, **j**, **k** inset) and mean ± s.e.m. (**h**, **i**, **k**). Unpaired Student’s *t* test with 95% confidence levels two-tailed (**a**, **j**). Ratio paired Student’s *t* test with 95% confidence levels two-tailed (**b**, **d**, **f**, **g**), Mann–Whitney test (**h**, **i**, **k** insets). The gating strategy for **a** is shown in Supplementary Fig. 8a, gating strategy for **j** is shown in Supplementary Fig. 2b.

SLAMF5 was previously shown to regulate the survival of CLL cells, which express high levels of this receptor^31^. Since Bregs express high levels of SLAMF5, we tested whether blocking this receptor interferes with survival signaling in Bregs, as well. To specifically follow the role of SLAMF5 expressed on B cells, we blocked its activity in purified B-cell populations. B cells from EAE-induced WT mice in remission were cultured for 48 h under IL-10-inducing conditions (LPS) with either isotype control or SLAMF5-blocking antibody^30–32^. Blocking SLAMF5 significantly increased Breg numbers (Fig. 4a) compared to control-IgG. The increase of Breg numbers was not dependent on proliferation (Fig. 4b), but due to an increase of Breg survival (Fig. 4c, d). While the Breg live-population was increased, non-Bregs were not affected, suggesting that SLAMF5 specifically suppresses pro-survival signaling in Bregs (Fig. 4e, f).

To further analyze SLAMF5-dependent pathways in Breg populations, we sorted Bregs from the IL-10 GFP Vert-x reporter mice. Sorted Bregs (CD19^+^GFP^hi^) were treated for 24 h with SLAMF5-blocking or control-IgG antibodies and were analyzed for the mRNA levels of Bcl-2 family members. Increased levels of *Bcl-2* and *Bcl-xl* (Fig. 4g) were detected in the SLAMF5-blocked population, suggesting that SLAMF5 restricts Breg numbers via survival signaling pathways.

### Blocking of SLAMF5 mitigates EAE and increases Breg levels

We next examined the effect of SLAMF5-blocking in vivo on EAE pathogenesis. WT mice were induced with MOG_35-55_, and treated with SLAMF5-blocking or IgG-control antibodies, starting on day 7 after disease induction. Injections were repeated on days 9 and 11, and mice were followed through day 15 (Fig. 4h). Clinical signs of disease appeared in both groups 10–11 days after disease induction, reaching a maximal mean clinical score of 3.45 ± 0.134 for the IgG-treated group, at day 15 when disease incidence was 100%. The SLAMF5-blocking-treated group reached a maximal mean score of 2.72 ± 0.195, and an incidence of 94%. The difference between the two groups was significant, as indicated by the AUC (Fig. 4h, AUC in the inset, Supplementary Fig. 8b). This was accompanied by significantly reduced weight loss in mice treated with SLAMF5-blocking antibody (Fig. 4i, AUC in inset). Analysis of splenic Breg populations at the end of the experiment showed that blocking SLAMF5 in EAE-induced (Fig. 4j) or healthy mice (Supplementary Fig. 8c) upregulated the levels of total Bregs and the Breg subpopulations T2MzP and CD138^+^ (Fig. 4j), suggesting that SLAMF5-blocking downregulates Breg numbers in health and disease.

Since SLAMF5 is expressed on several immune cells, its blockade can affect the function of non-B cells. To determine whether the effect of SLAMF5-blocking may also stem from an effect on the non-B-cell populations, EAE-induced JHT mice were treated with SLAMF5-blocking or IgG antibodies. Clinical signs of disease appeared in both treatment groups 7–8 days after disease induction, and both groups reached a similar maximal mean clinical score of 3.27 ± 0.104 for the IgG-treated group and 3.36 ± 0.07 for the anti-SLAMF5-treated group. In both groups, a disease incidence of 100% was reached. Overall, no significant differences were observed over the disease course (Fig. 4k and AUC in the inset, Supplementary Fig. 8d). These results suggest that blocking of SLAMF5 leads to an improved clinical score and outcome for EAE mice by boosting their regulatory B-cell levels and that the beneficial effect of SLAMF5-blocking is mediated mainly through its effect on B cells.

### Blocking SLAMF5 increases c-Maf

**Fig. 5:**
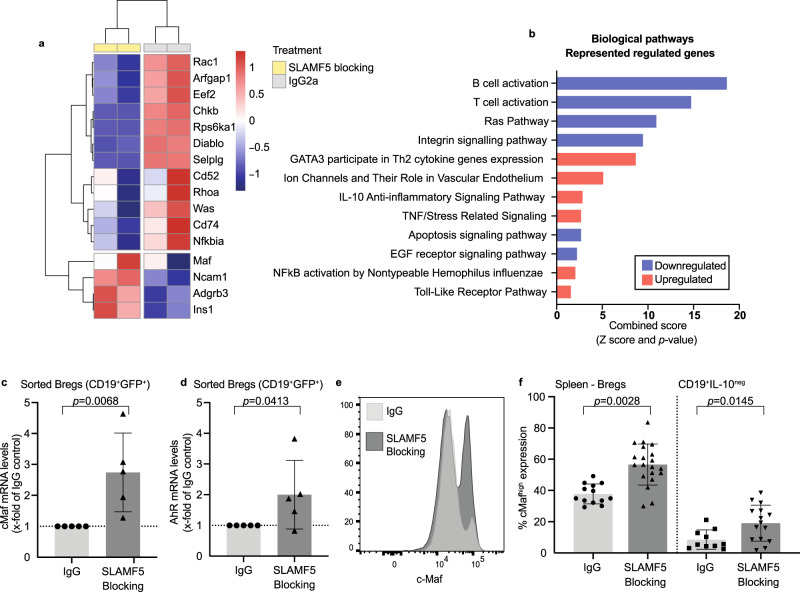
SLAMF5 regulates the transcription factor c-Maf. B cells derived from EAE-induced Vert-x mice were sorted for Bregs (CD19^+^GFP^+^) and treated for 24 h with SLAMF5-blocking or IgG-control antibodies. RNA was then extracted from the cells and analyzed. **a**, **b** mRNA-seq analysis of differentially expressed genes in anti-SLAMF5 compared to IgG-treated cells performed in two independent replicates. **a** Heatmap showing differentially expressed genes, with the log2 normalized counts standardized for each gene to a mean of zero. The hierarchical clustering of the genes is shown. The expression profile is accompanied by a colored bar indicating the standardized log2 normalized counts. Blue: downregulated; red: upregulated genes. **b** Pathway enrichment analysis of 277 genes that were found to be significantly changed (absolute change > 2; *P* value < 0.05) was done using the EnrichR platform; the graph shows upregulated (red) and downregulated (blue) pathways (IgG *n* = 2; SLAMF5-blocking *n* = 2, each sample is a pool of cells derived of three mice). **c** qPCR validation analysis of c-Maf mRNA in SLAMF5-blocked Vert-x sorted Bregs (CD19^+^GFP^+^) (IgG *n* = 5; SLAMF5-blocking *n* = 5. Each dot represents a pool of two to three mice, four independent experiments). **d** AhR mRNA levels in Vert-x sorted Bregs following SLAMF5-blocking. Data are shown as *x*-fold of the treatment group compared to IgG-control (IgG *n* = 5; SLAMF5-blocking *n* = 5. Each dot represents a pool of two to three mice, four independent experiments). **e**, **f** EAE was induced in WT mice. On days 7, 9, and 11, the mice were injected i.v. with 30 µg SLAMF5-blocking Ab or IgG. On day 15, spleens were collected and analyzed for c-Maf expression by FACS. **e** Representative histogram showing c-Maf^high^ expression of Bregs or non-Bregs. **f** Bar chart showing c-Maf levels in Bregs and non-Bregs. (non-Bregs: IgG n = 10; SLAMF5-blocking *n* = 15, Bregs: IgG *n* = 5; SLAMF5-blocking *n* = 10, two independent experiments). C-Maf expression was done under the Breg or non-Breg gate as shown in Supplementary Fig. 2c. Data expressed as mean ± s.d. (**c**, **d**, **f**). Ratio paired Student’s *t* test with 95% confidence levels two-tailed (**c**, **d**), unpaired Student’s *t* test with 95% confidence levels two-tailed (**f**). Pathway analysis was done using EnrichR and showing combined score (combined = ln(*P* value) x z-score. *P* value is derived from Fisher’s exact test, z-score is derived of a modified Fisher exact test.

To attain a deeper understanding of the role of SLAMF5 in Bregs, total cellular mRNA levels in SLAMF5-blocked Bregs were analyzed by RNA-Seq. Sorted Bregs of EAE-induced Vert-x mice (Supplementary Fig. 8e) were incubated with the SLAMF5-blocking or IgG antibodies. After 24 h, mRNA was extracted, prepared for sequencing, and analyzed. Significant changes (absolute change > 2; *P* value < 0.05) in 277 genes were found, with 141 genes downregulated and 136 genes upregulated compared to the IgG-control group. The differential regulation is summarized in Fig. 5a. Analysis using the EnrichR analysis software identified various pathways that were enriched in the treatment group (Fig. 5a, b). Blocking SLAMF5 resulted in changes associated with cell survival, e.g., apoptosis; NF-κB pathways, IL-10 signaling pathways, e.g., T-cell activation; IL-10 anti-inflammatory pathways, and others (Fig. 5a, b).

We chose to focus on the *maf* gene, encoding the transcription factor (TF) c-Maf. This TF is essential for IL-10 production in T cells and macrophages^47^ and was recently shown to be crucial for IL-10 production in regulatory B cells^48^. *Maf* expression was dramatically increased in one of the RNA-seq samples (Fig. 5a). This phenotype was further validated using qPCR, confirming that blocking of SLAMF5 in vitro increases the expression of this essential TF (Fig. 5c). We further analyzed the c-Maf co-TF AhR^49^, which was recently shown to be essential for IL-10 production in Bregs^50^, and found that its mRNA levels were elevated under SLAMF5-blocking conditions (Fig. 5d).

We next followed the in vivo regulation of c-Maf expression by SLAMF5. EAE-induced mice were treated with SLAMF5-blocking or IgG-control antibodies on days 7, 9, and 11 following disease induction. Consistent with the in vitro data, increased levels of c-Maf protein were detected in the regulatory B cells of the mice treated with SLAMF5-blocking antibody (Fig. 5e, f). This increase was specific to the EAE-derived Bregs and was not observed in the IL-10^neg^ B-cell population (Fig. 5f). Furthermore, Bregs and non-Bregs derived from naive mice treated with SLAMF5-blocking antibody did not show any change in their c-Maf levels (Supplementary Fig. 8e), indicating that the effect of SLAMF5-blocking on c-Maf is specific to cells derived from the diseased mice, and suggesting that SLAMF5 negatively regulates c-Maf and AhR expression during EAE.

### SLAMF5 regulates human Breg survival and c-Maf expression

**Fig. 6:**
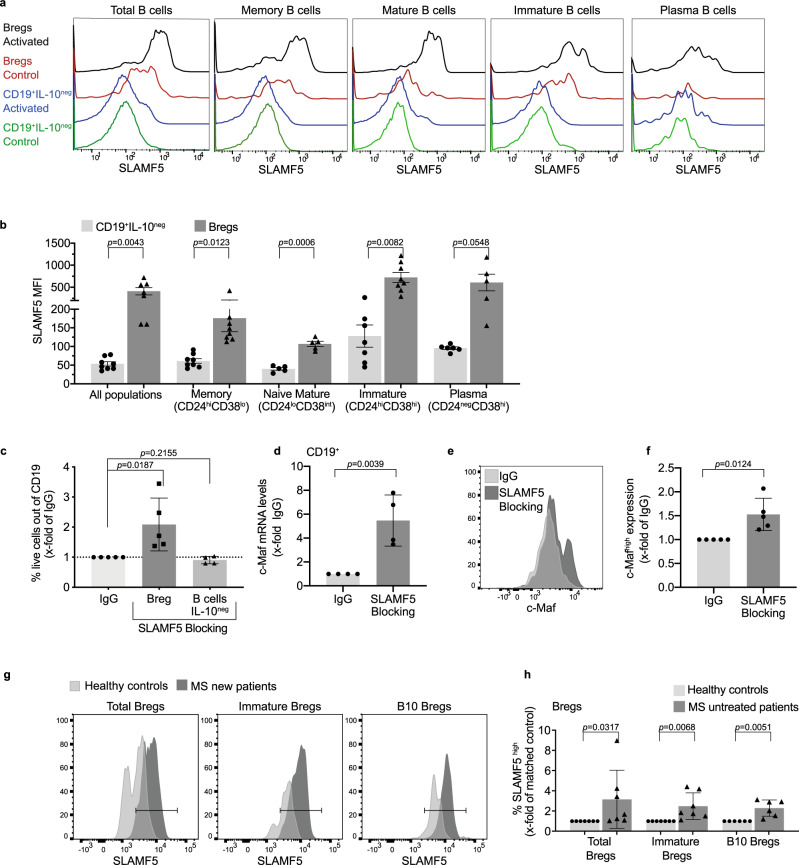
SLAMF5 fine-tunes the survival of human Bregs. **a**, **b** Healthy human PBMCs were activated for 5 h with PMA, ionomycin, brefeldin A, and monensin or with monensin alone (control) and analyzed by FACS for SLAMF5 expression on B-cell populations: total B cells: CD19^+^; memory B cells: CD19^+^CD24^high^CD38^low^; naive mature B cells: CD19^+^CD24^low^CD38 ^low^; immature B cells: CD19^+^CD24^high^CD38^high^; and plasma B cells: CD19^+^CD24^low^CD38^high^. Regulatory B-cell subpopulations were analyzed with similar markers under the CD19^+^IL-10 gate. Gating is shown in Supplementary Fig. 9a. **a** Representative histograms, and (**b**) bar charts of SLAMF5 expression on IL-10^+^ (Breg) and IL-10^neg^ (non-Breg) populations. (non-Bregs *n* = 7; Bregs *n* = 7; memory B cells *n* = 8; memory Bregs *n* = 7; naive B cells *n* = 5; naive Bregs *n* = 5; transitional B cells *n* = 7; transitional Breg *n* = 8; plasma B cell *n* = 6; plasma Breg *n* = 5, four independent experiments). **c**–**f** Purified human B cells from healthy donors were treated with SLAMF5-blocking or IgG-control antibody for 48 h. For the last 5 h, the cells were activated with PIM and analyzed for (**c**) cell survival by Zombie dye staining. Bars chart of live Bregs (CD19^+^IL-10^+^Zombie^neg^) or non-Breg B cells (CD19^+^IL-10^−^Zombie^−^) out of total CD19^+^. (IgG: Breg *n* = 5; non-Breg = 5, SLAMF5-blocking: Breg *n* = 5; non-Breg *n* = 5, three independent experiments). **d** After 24 h of treatment, cells were collected for mRNA analysis of c-Maf on total B cells. Results are shown as *x*-fold of treatment compared to IgG-control. (IgG *n* = 4; SLAMF5-blocking *n* = 4, two independent experiments). **e**, **f** c-Maf expression in Bregs was analyzed by FACS after 48 h. **e** Representative histogram, and (**f**) bar chart of c-Maf expression. Results are shown as *x*-fold of treatment compared to IgG-control. (IgG = 5; SLAMF5-blocking *n* = 5, two independent experiments). **g**, **h** Blood samples derived from newly diagnosed untreated multiple sclerosis patients and matched healthy controls were processed, and PBMC were activated for 5 h with PMA, ionomycin, monensin, and brefeldin A, and stained for SLAMF5 and IL-10 on Breg populations. Total Bregs: CD19^+^IL-10^+^; immature Bregs: CD19^+^IL-10^+^CD24^+^CD38^+^; B10 Bregs: CD19^+^IL-10^+^, CD24^+^CD27^+^. **g** Representative histograms showing the gate used to define SLAMF5^high^ expression and **h** bar charts of SLAMF5^high^ expression on Breg populations presented as fold increase over each sex- and age-matched control. Data expressed as mean ± s.e.m. (**b**) or mean ± s.d. (**c**, **d**, **f**, **h**). Paired Student’s *t* test with 95% confidence levels two-tailed (**b**). Ratio paired Student’s *t* test with 95% confidence levels two-tailed unpaired Student’s *t* test with 95% confidence levels two-tailed (**c**, **d**, **f**, **h**). The gating strategy for **a** and **b** is shown in Supplementary Fig. 9a, gating strategy for **c** is shown in Supplementary Fig. 8a, gating strategy for **g** and **h** is shown in Supplementary Fig. 9b.

We next wished to determine whether SLAMF5 plays a regulatory role in human Bregs. Analysis of SLAMF5 expression on Bregs derived from the peripheral blood (PB) of healthy donors revealed that human Bregs express high levels of this receptor compared to IL-10-negative B cells (Fig. 6a, b). To determine whether SLAMF5 plays a role in human Breg survival, human PB B cells from healthy donors were treated with anti-SLAMF5-blocking or IgG-control antibodies. Blocking SLAMF5 significantly increased the live-Breg population, while showing no effect on the IL-10-negative B cells (Fig. 6c). Furthermore, an increase in c-Maf was detected in mRNA in total B cells (Fig. 6d) and protein (Fig. 6e, f) levels in Bregs incubated with SLAMF5-blocking antibody.

Multiple sclerosis (MS) patients were shown to have functionally impaired Breg populations^51^. In addition, there is evidence that MS patients exhibit reduced numbers of Bregs^52–54^. Since our results indicate a negative regulatory mechanism on Breg numbers by SLAMF5, we determined whether SLAMF5 expression is altered on Bregs derived from newly diagnosed untreated MS patients. Analysis of SLAMF5 revealed a significant increase of its expression on total Bregs (CD19^+^IL-10^+^), immature Bregs (also termed immature transitional Bregs, CD19^+^CD24^+^CD38^hi^IL-10^+^)^55^, and B10 Bregs (CD19^+^CD24^+^CD27^+^IL-10^+^)^56^ compared to healthy donors (Fig. 6g, h). These results suggest that dysregulation of SLAMF5 expression on Bregs may lead to abnormal Breg numbers and function.

## Discussion

Regulatory B cells are important suppressors of inflammation, and their homeostasis must be tightly controlled. Overactivation of regulatory cells may lead to immune deficiencies and susceptibility to pathogens and cancer, while insufficient activation may lead to an uncontrolled immune response and autoimmune diseases^57^. In this study, we identified a fine-tuning mechanism that negatively regulates Breg number and function through the cell surface receptor SLAMF5 in humans and in mice. Our results show that SLAMF5 deficiency significantly mitigates EAE. *slamf5*^*−/−*^ mice show a delayed onset of EAE, and significantly milder disease with lower incidence compared to WT mice.

Bregs have a protective role in EAE^58^, and their numbers and their suppressive functions are increased during the inflammatory phase of autoimmune diseases. During EAE, in the absence of SLAMF5, mice exhibit higher levels of Breg both in the SC and lymphoid organs. Specifically, in the periphery, we show an increase in the accumulation of T2MzP and CD138 + Breg subpopulations. Treating EAE-induced WT mice with SLAMF5-blocking Ab of increased Breg levels and disease mitigation. These findings indicate that SLAMF5 which is highly expressed on Bregs fine-tunes Breg numbers.

SLAM receptors mediate activating-or inhibiting-signals, depending on the cell type and presence of various downstream adaptors. Blocking SLAMF5 leads to an increased expression of anti-apoptotic genes of the BCL-2 family, consequently increasing Breg survival. Our previous results showed that blocking the SLAMF5 receptor expressed on CLL cells leads to a decrease in cell viability, suggesting that the effect of SLAMF5 is cell and disease-specific^31^. In addition, SLAMF5 controls the expression of the *maf* gene, which encodes the protein c-Maf. c-Maf is a DNA-binding transcription factor that can act as a transcriptional activator or repressor, depending on the binding site and binding partner^47^. c-Maf was recently found to be crucial for IL-10 production in regulatory B cells^48^. It regulates IL-10 with its co-factor AhR, which was also recently shown to be crucial for IL-10 production in Bregs^50^. We demonstrate that c-Maf and Ahr expression levels are elevated following SLAMF5-blocking. Thus, SLAMF5 controls both the survival of Bregs through the BCL family members and the potential of B cells to differentiate into Bregs by regulating the expression of c-Maf and its co-factor, AhR. We suggest that c-Maf might have a direct role in regulating Breg survival by controlling BCL2 expression. Alternatively, its function in these cells might not be related to cell survival, but only to the regulation of IL-10 expression.

While SLAMF5 inhibition showed no effect on the in vitro survival of IL-10-negative B cells, its in vivo effect on these cells cannot be completely excluded. However, since in the MOG_35-55_ model, B cells do not secrete disease-associated antibodies or pro-inflammatory cytokines or act as strong APCs, we suggest that the effect of SLAM5 is attributed mainly to the regulatory B cells.

In MS, the B-cell cytokine profile shifts toward a more pro-inflammatory one, and accordingly, patients exhibit various Breg defects such as irregular numbers^52,53,59^ and impaired function^60–62^. Regulatory B cells of naive, newly diagnosed and untreated, MS patients express significantly higher levels of SLAMF5 compared to its expression on healthy controls, suggesting a role for SLAMF5 as a negative regulator of Breg levels and activity in the disease. Our results imply that the abnormal increase of SLAMF5 on Bregs derived from untreated MS patients may induce an excessive negative regulation of these cells and may underlie the insufficient Breg response during MS.

In conclusion, we suggest that SLAMF5 can serve as a therapeutic target in MS and other autoimmune diseases. Blocking SLAMF5 can increase the survival of Bregs in these patients, while not affecting the pro-inflammatory B cells. As deficiency of SLAMF5 has almost no effect on the immune repertoire and response in *slamf5*^−/−^ mice^29^, it is likely that SLAMF5 can offer a safe immune-target to boost self-regulatory mechanisms to reduce autoimmunity in patients.

## Methods

### Mice

C57BL/6 WT (C57BL/6JOlaHsd, Envigo, Israel), C57BL/6 (CD45.1), C57BL/6 SLAMF5-deficient^29^, JHT-deficient^42^, Vert-x (IL-10 GFP Reporter)^45^, and C57BL/6 SLAMF6-deficient^63^ mice were used at 6–12 weeks of age. Both females and males were used, and the groups where age- and sex-matched in each experiment, control mice were bred separately. All animal procedures were approved by the Animal Research Committee at the Weizmann Institute of Science. Mice were housed in an SPF animal facility, with an ambient temperature of 22 ± 1°C in a light/dark cycle of 12 /12 h with 50 ± 5% humidity. Mice were euthanized using carbon dioxide (CO_2_) overdose.

### Experimental autoimmune encephalomyelitis Induction

Mice were injected subcutaneously with 200 μg MOG_35-55,_ (synthesized by Genscript) in incomplete Freund’s adjuvant supplemented with 3 mg ml^−1^ heat‐inactivated Mycobacterium tuberculosis (Sigma-Aldrich). Pertussis toxin (Sigma-Aldrich), 200 ng per mouse, was injected I.P. immediately after the MOG_35-55_ injection, and again after 48 h^64^.

Mice were examined daily and scored by a researcher blinded to the experimental treatment of the animals using the following scoring system: 0—no disease, 1—loss of tail tonicity, 2— hind leg weakness, 3—complete hind leg paralysis, 3.5—complete hind leg paralysis with partial hind body paralysis, 4—full hind and foreleg paralysis, and 5—moribund or dead animals^64^.

For EAE experiments, both males and females were used at ages 9–12 weeks, where age and sex-matched controls were used in each experiment.

### Human peripheral blood lymphocyte preparation

Peripheral blood samples of MS patients and age- and sex-matched healthy controls were provided in compliance with the Institutional Review Board of the Barzilai Medical Center, Ashkelon, Israel. Consent was informed, and the samples were obtained by written consent. The Weizmann institute review board approved the study: IRB number 1339-1. Patient characteristics are listed in Supplementary Table 1. Peripheral blood samples of healthy controls were provided by the “Magen David Adom in Israel” blood bank. Peripheral blood mononuclear cells (PBMCs) homogenates were obtained by centrifuging the samples at 1400× *g* 5 min in order to separate the buffy coat, which was then treated with BD Pharm Lyse™ (BD Biosciences).

### Mouse cell preparation

Mouse spleens and draining lymph nodes (LNs, including the axillary, brachial, inguinal, lumbar, and caudal LNs) were dissected postmortem and collected in PBS (Biological Industries) with 2% FCS. Briefly, cell homogenate was prepared, treated with red blood lysis buffer. Cells were then sashed and strained through 40-µm nylon mesh and counted. Spinal cords were dissected postmortem into RPMI, by flushing the spinal cord out of the spine with RPMI (Gibco) using an 18-gauge needle. Due to the low lymphocyte yield from CNS tissue, four to five spinal cords were pooled for each measurement. Infiltrating leukocytes were prepared in a two separation gradients method; spinal cords were then gently homogenized using a glass tissue grinder in 5 ml RPMI. Homogenate was added to a tube containing a 10 ml RPMI, 9 ml Percoll, and 1 ml 10× PBS. Homogenate was then centrifuged at 7800×*g*. for 30 min at RT. Leukocytes were then collected from the middle fraction of the gradient, strained through a 40-µm nylon mesh, and washed with PBS. Leukocytes were then resuspended in 1 ml buffer (1% bovine serum albumin, 0.02% sodium azide in calcium and magnesium-free PBS) and underlay with 1 ml ficoll. Samples were then centrifuged at 1400×*g* for 25 min, RT, no brake. The Middle white layer, which contains the leukocytes, was then removed, washed, and counted^65^.

### Flow cytometry staining

Flow cytometry (FACS) analysis was performed using FACS Canto (BD Biosciences) and data were collected using FACSDIva8 (BD Biosciences). FACS data analysis was done using Flowjo v10. Antibodies are listed in Supplementary Table 2.

Intracellular cytokine analysis of T-cell cytokines and transcription was done following 24 h cultures of 5 × 10^6^ cells per ml in 10% serum IMEM medium with 10 μg ml^−1^ MOG35-55. For the last 5 h of the culture PMA (100 ng ml^−1^, Sigma-Aldrich), ionomycin (1 μg ml^−1^; Sigma-Aldrich), monensin, and brefeldin A (1:1000, Biolegend) were added.

For detection of IL-10 in B cells, B cells were cultured at 2.5 × 10^6^ cells per ml in 10% serum IMEM with PMA (100 ng ml^−1^, Sigma-Aldrich), ionomycin (1 μg ml^−1^; Sigma-Aldrich), and monensin (1:1000, Biolegend) for 5 h (use of PMA, ionomycin, and monensin is abbreviated as PIM). Cells were then stained with surface markers followed by fixation, permeabilization, and incubation with intracellular Abs. For transcription factors, staining fixation was done using a transcription factor fixation/permeabilization kit (Invitrogen), for cytokine staining fixation was done using BD Cytofix/Cytoperm (BD Biosciences).

### Chimeric mice

CD45.1 C57BL/6 WT mice aged 6 weeks were lethally irradiated with 850 rad, X-ray (XRAD 320, PXI). After 24 h, mice were injected with 2 × 10^6^ BM cells consisting of 80% JHT-deficient BM and 20% WT or SLAMF5-deficient BM. After BM reconstitution (8–10 weeks), chimeric mice were induced with EAE using a milder protocol. Mice were injected with 150 μg MOG_35-55_ in incomplete Freund’s adjuvant supplemented with 3 mg ml^−1^ heat‐inactivated Mycobacterium tuberculosis (Sigma-Aldrich). Pertussis toxin, 50 ng per mouse, was injected I.P. immediately after the MOG_35-55_ injection, and again after 48 h. For proliferation staining, B cells were treated with the cell proliferation dye (CPD) (eBiosceince) according to the manufacturer’s instructions.

### In vitro Breg generation assay

Spleens of naive mice were collected postmortem. B cells were purified by positive B-cell selection with B220 magnetic beads (BD Biosciences). B cells were then incubated at a concentration of 2.5 × 10^6^ per ml for 24 or 72 h in full IMEM with 10 µg ml^−1^ Lipopolysaccharide (LPS L2880, Sigma-Aldrich).

### SLAMF5 blocking—in vitro

#### Mouse cells

B cells were isolated from WT, *slamf5*^−/−^ or Vert-x mice in EAE remission or healthy naive WT, and incubated at a concentration of 2.5 × 10^6^ cells per ml for 24 or 48 h in the presence of 5 µg ml^−1^ LPS (Sigma) and 30 μg ml^−1^ SLAMF5-blocking Ab^30,31^ or isotype control-IgG2a (MG2a-53, BioLegend), as previously described^30,31^.

#### Human cells

B cells were isolated from PB derived from healthy donors by B cell-negative selection with EasySep™ Human B Cell Isolation Kit (Stemcell Technologies). B cells were then incubated at a concentration of 2.5 × 10^6^ per ml for 24 or 48 h in the presence of 30 μg ml^−1^ SLAMF5-blocking Ab (B4)^30,31^ or isotype control-IgG2a (BioLegend).

### Real-time reverse transcription–PCR analysis

RNA extraction was done using Direct-zol RNA microprep kit (Zymo Research). Reverse transcription was performed by Qiagen RT kit according to the manufacturer’s instructions. qPCR was performed using a Light-Cycler 480 Instrument (Roche Diagnostics) and Light-Cycler HotStart DNA SYBR Green I mix kit (Roche Diagnostics). Primers used are listed in Supplementary Table 3.

### RNA sequencing

For RNA preparation, 3 × 10^5^ CD19^+^GFP^+^ cells were sorted (using BD FACSMelody) from 2-3 EAE-induced Vert-x mice. Cells were incubated with SLAMF5-blocking or IgG-control Ab for 24 h. Then the total RNA was extracted from the cells using the Direct-zol RNA kit (Zymo Research) according to the manufacturer’s instructions. A bulk adaptation of the MARS-Seq protocol^66^ was utilized to generate the RNA-Seq libraries for expression profiling of both treatment groups. Briefly, RNA from each sample was barcoded during reverse transcription and then pooled. Subsequently, after cleanup using Agencourt Ampure XP beads (Beckman Coulter), the pooled samples were subjected to second-strand synthesis and were amplified linearly by T7 in vitro transcription. The resulting RNA was thereafter fragmented, tagged with Illumina sequences during ligation, and subjected to RT and PCR resulting in a sequencing-ready library. Libraries were quantified by Qubit, TapeStation, and qPCR, as previously described^66^. Sequencing was done using a Nextseq 75 cycle high output kit (Illumina; paired-end sequencing).

Analysis of the MARS-seq was done using the UTAP pipeline (the Weizmann Institute Bioinformatics Unit) to map the reads to the mouse genome and to calculate Unique Molecule Identifier (UMI) counts per gene. Reads were trimmed using cutadapt (which removes adapter sequences from high throughput sequencing reads; with parameters: -a AGATCGGAAGAGCACACGTCTGAACTCCAGTCAC -a “A{10}” –times 2 -u 3 -u −3 -q 20 -m 25). Reads were mapped to the (Mus_Musculus, UCSC, mm10) genome, STAR v2.4.2a. Genes having a minimum of five reads in at least one sample were considered for further analysis^67^. Differentially expressed gene (DEG) detection and count normalization analysis were performed by DESeq2^68^. *P* values in the UTAP results were adjusted for multiple testing using the Benjamini and Hochberg procedure; false discovery rate (FDR) correction was done by *fdrtool*^69^.

### Breg T-cell suppression assay

T-cell suppression assay by regulatory B cells was performed^70^. Briefly, WT or *slamf5*^−/−^ B cells were first purified by B220^+^ magnetic beads and then transitional 2 (T2) B cells were isolated by sorting from spleens of mice in remission from EAE, and cultured 1:1 or 2:1 with WT or *slamf5*^−/−^ CD4^+^CD25^−^ T cells isolated by sorting for 72 h in the presence of 1 μg ml^−1^ anti-CD3 (17A2; Biolegend) and 3 mg ml^−1^ anti-CD28 (3751; Biolegend). To neutralize IL-10, cells were incubated with 10 μg ml^−1^ of mouse IL-10 antibody (MAB417, R&D systems) or with IgG1-control (Biolegend).

### Statistics

Statistical analysis was performed using GraphPad Prism 8 software (San Diego, California, USA). Disease scoring and weight loss graphs show s.e.m. The significance of disease score and weight loss was analyzed by calculating the area under the curve parameter (AUC). AUC analysis was performed using the Mann–Whitney test. Experiments analyzing peripheral leukocytes from EAE-induced mice included all mice with all scores. Experiments analyzing CNS leukocytes from EAE-induced mice included only mice of score >= 2. When pooled samples were required (e.g., spinal cord analysis) all mice had the same score. The SLAMF5-blocking assay was analyzed using a two-tailed paired *t* test; all other analyses were performed using one- or two-tailed unpaired *t* test as appropriate. In the bar graphs, data represent mean ± s.d., and each dot represents one biological repeat (mouse or human) unless specified otherwise.

### Reporting summary

Further information on research design is available in the Nature Research Reporting Summary linked to this article.

## Supplementary information

Supplementary Information

Reporting Summary

## Data Availability

The RNA sequencing data discussed in this publication have been deposited in NCBI’s Gene Expression Omnibus and are accessible through GEO Series accession number GSE160828. All other data are included in the Supplementary Information or available from the authors upon reasonable requests, as are unique reagents used in this Article. Source data are provided with this paper.

## Source data

Source Data
