# Supplementary material for: The survival and function of IL-10-producing regulatory B cells are negatively controlled by SLAMF5
Source: Nat Commun. 2021 Mar 25;12:1893. doi: 10.1038/s41467-021-22230-z (PMC7994628; doi:10.1038/s41467-021-22230-z)
Supplement: Supplementary file 1 — Supplementary Information [file 41467_2021_22230_MOESM1_ESM.pdf]

Supplementary Information

Title: The survival and function of IL-10-producing regulatory B cells are negatively controlled by SLAMF5

Authors: Radomir Lihi[1], Kramer P. Matthias[1],Perpinial Michal[1], Schottlender Nofar[1], Rabani Stav[1], David Keren[1], Wiener Anna[1], Lewinsky Hadas[1], Becker-Herman Shirly[1], Aharoni Rina[1], Milo Ron[2] , Mauri Claudia[3], Shachar Idit[1].

Affiliations:

1. Department of Immunology, The Weizmann Institute of Science, Rehovot, Israel
2. Department of Neurology, Barzilai University Medical Center, Ashkelon; Faculty of Health Sciences, Ben-Gurion University of the Negev, Beer-Sheva
3. Centre for Rheumatology Research, Department of Medicine, University College London, UK

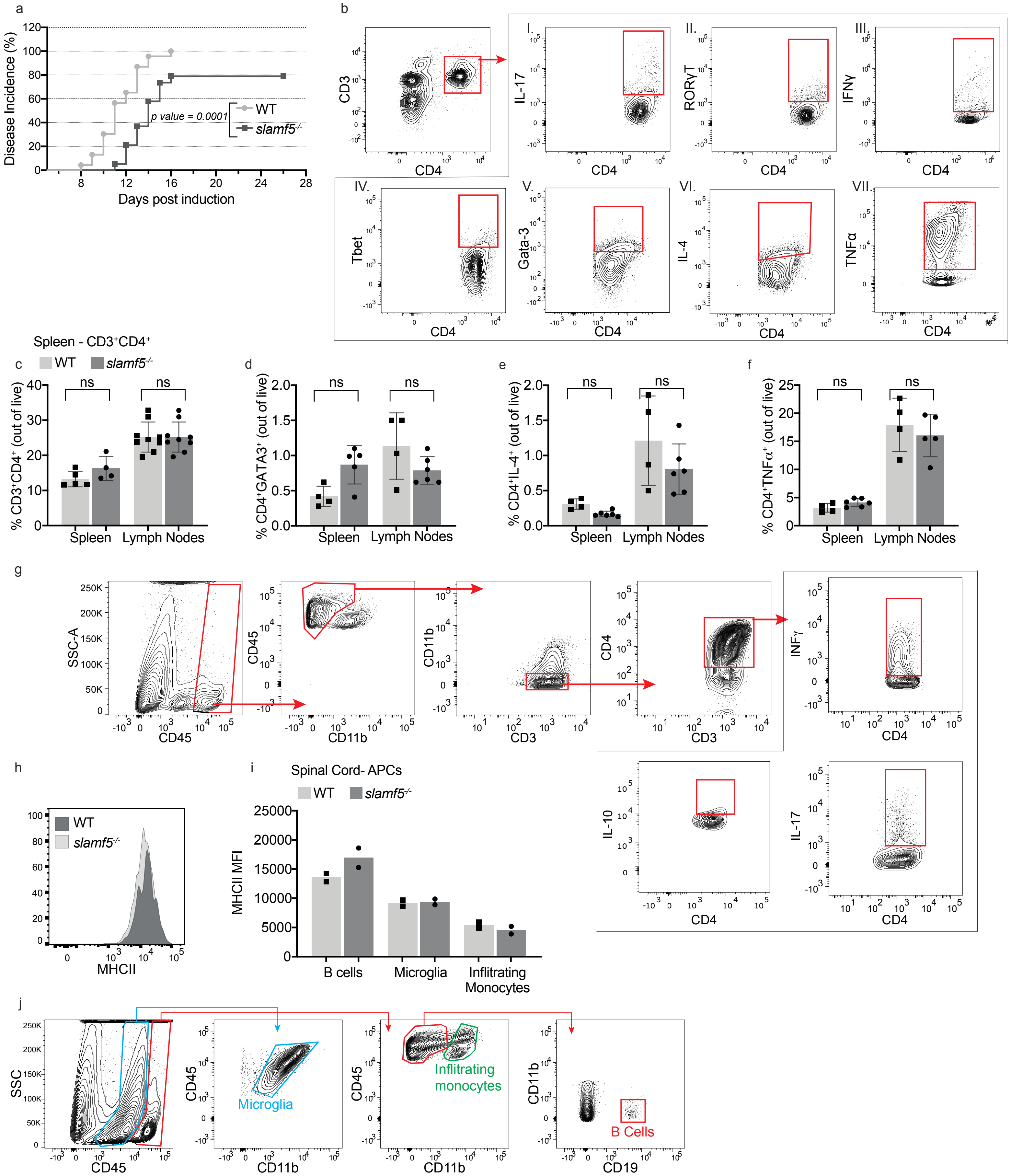

Supplementary Figure 1 – SLAMF5 deficiency protects mice from EAE

WT and slamf5$^{-/-}$ mice were induced for EAE (MOG$_{35-55}$). (**a**) Graph depicting the disease incidence of WT or slamf5$^{-/-}$ mice. Mice were considered sick with a score =>1 (WT n= 23; slamf5$^{-/-}$ n=19, three independent experiments). (**b-f**) On day 15, spleens and LNs were analyzed for expression of transcription factors and cytokines by CD4^{+} T cells, all under the CD4^{+}CD3^{+} gate. (**b**) showing gating strategy used in the following figures: I.- IV. In Figure 1c-d; I.,III. In Figure 3h-i; V.-VII. In Supplementary Figure d-f. (**c**) Total CD3^{+}CD4^{+} (WT spleen n=5; WT LNs n=4; slamf5$^{-/-}$ spleen n= 9; slamf5$^{-/-}$ LNs n=9, one experiment), (**d**) IL-4^{+} (WT spleen n=4; WT LNs n=5; slamf5$^{-/-}$ spleen n=4; slamf5$^{-/-}$ LNs n=6, one experiment), (**e**) Gata3^{+} (WT spleen n=4; WT LNs n=6; slamf5$^{-/-}$ spleen n=4; slamf5$^{-/-}$ LNs n=6, one experiment), (**f**) TNFα^{+} (WT spleen n=4; WT LNs n=6; slamf5$^{-/-}$ spleen n=4; slamf5$^{-/-}$ LNs n=5, one experiment). (**g**) Gating strategy used in Figure 1 e,h,k. (**h**) Representative histogram for MHCII on B cells in the spinal cord (gating strategy shown in Supplementary figure 2a) . (**i**) MHCII expression on B cells (CD45^{hi}CD11b^{-}CD19^{+}), microglia (CD45^{int}CD11b^{+}), and infiltrating monocytes (CD45^{hi}, CD11b^{+}). In the spinal cords, each dot represents a pool of 4-5 mice with similar disease score (WT n=2; slamf5$^{-/-}$ n=2, one experiment). (**j**) gating strategy as used for Supplementary Figure 1 h-i. Data expressed as mean ± s.d (b-e, g). Log-rank (Mantel-Cox) test (a). Unpaired Student's t-test with 95% confidence levels two-tailed (c-f).

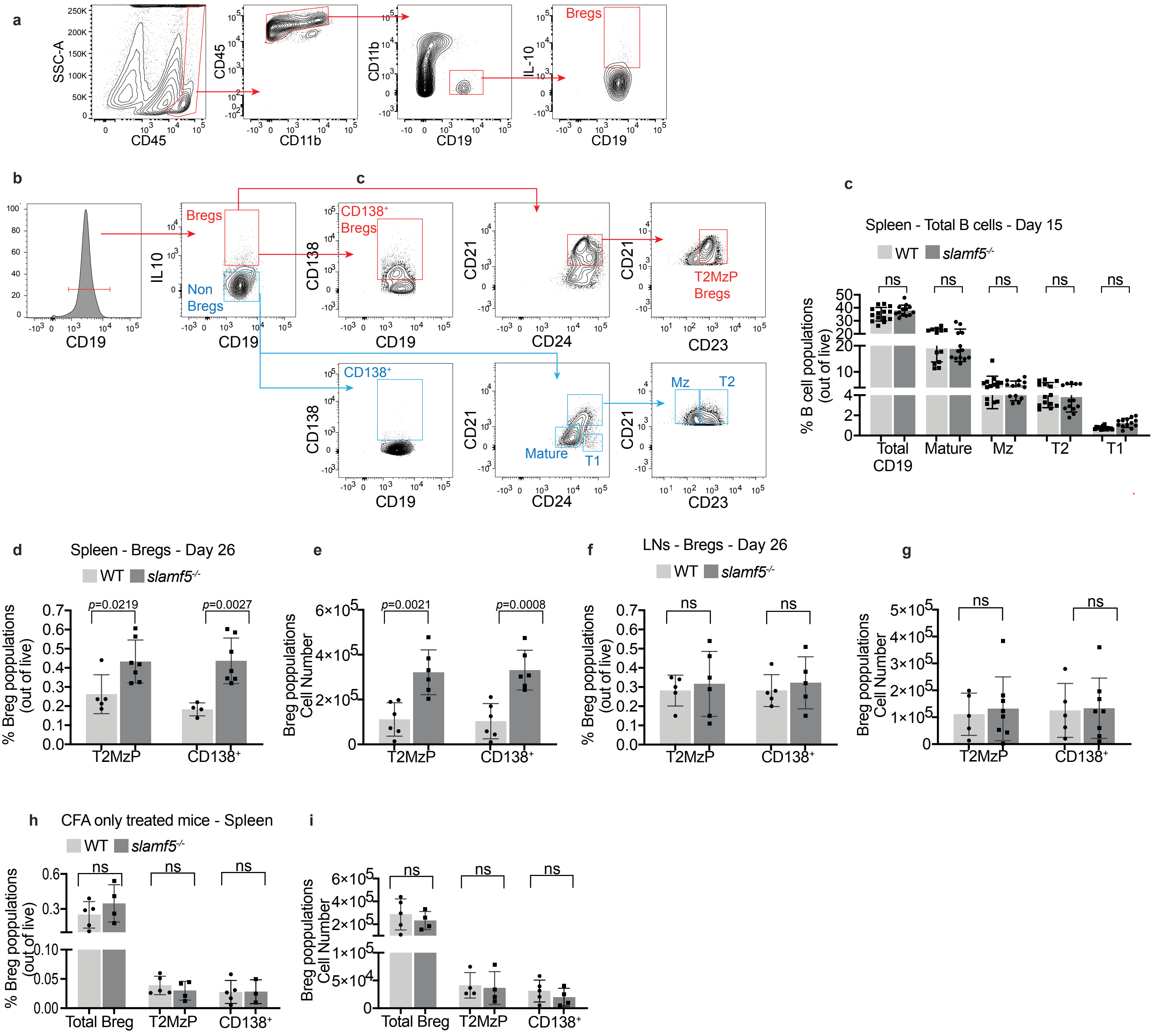

Supplementary Figure 2 – SLAMF5 deficiency regulates Bregs levels in EAE

(**a**) Gating strategy for Bregs in the spinal cord as shown in Figure 2a. (**b**) Gating strategy for Breg and non-Breg B cell subpopulations. Gating strategy as used in all figures showing T2MzP and CD138$^+$ Bregs and T2MzP and CD138$^+$ non-Bregs (IL-10^{neg}). (**c**) WT and slamf5$^{-/-}$ mice induced with EAE were harvested at day 15 and their spleens were analyzed for B cell populations: Total CD19$^+$: CD19$^+$ (WT n=14; slamf5$^{-/-}$ n=13); Mature: CD19$^+$CD24^{low}CD21^{low} (WT n=13; slamf5$^{-/-}$ n=13); Marginal Zone (Mz): CD19$^+$CD24$^+$CD21$^+$CD23$^-$ (WT n=14; slamf5$^{-/-}$ n=13); Transitional 2 (T2): CD19$^+$CD24$^+$CD21$^+$CD23$^+$ (WT n=14; slamf5$^{-/-}$ n=13); and Transitional 1 (T1): CD19$^+$CD24$^+$CD21$^-$ (WT n=14; slamf5$^{-/-}$ n=13, two independent experiments). (**d-g**) WT and slamf5$^{-/-}$ mice were induced for EAE. After 26 days, spleens and LNs were analyzed by flow cytometry for Breg subpopulations according the following markers; T2-MzP Bregs: CD19$^+$IL-10$^+$CD24$^+$CD21$^+$CD23$^+$; and CD138$^+$ Bregs: CD19$^+$IL-10$^+$CD138$^+$ (gating strategy shown in Supplementary Figure 2b). Showing spleen (**d**) percentages (T2MzP: WT n=5; slamf5$^{-/-}$ n=7; CD138$^+$: WT n=4; slamf5$^{-/-}$ n=7) and (**e**) cell numbers (T2MzP: WT n=6; slamf5$^{-/-}$ n=6; CD138$^+$: WT n=6; slamf5$^{-/-}$ n=6, one experiment) and lymph nodes (**f**) percentages (T2MzP: WT n=5; slamf5$^{-/-}$ n=6; CD138$^+$: WT n=5; slamf5$^{-/-}$ n=5) and (**g**) cell numbers (T2MzP: WT n=6; slamf5$^{-/-}$ n=6; CD138$^+$: WT n=6; slamf5$^{-/-}$ n=6, one experiment). (**h-i**) Mice were treated only with CFA. Spleens were harvested after 15 days and Breg populations were analyzed for (**h**) percentages (total: WT n=5; slamf5$^{-/-}$ n=4; T2MzP WT n=5; slamf5$^{-/-}$ n=4; CD138$^+$: WT n=5, slamf5$^{-/-}$ n=3) and (**i**) cell number (total: WT n=5; slamf5$^{-/-}$ n=4; T2MzP WT n=4; slamf5$^{-/-}$ n=4, CD138$^+$: WT n=5, slamf5$^{-/-}$ n=4, one experiment). Each dot represents a biological repeat. Data expressed as mean ± s.d (b, d-i). Unpaired Student's t-test with 95% confidence levels two-tailed (b, d-i).

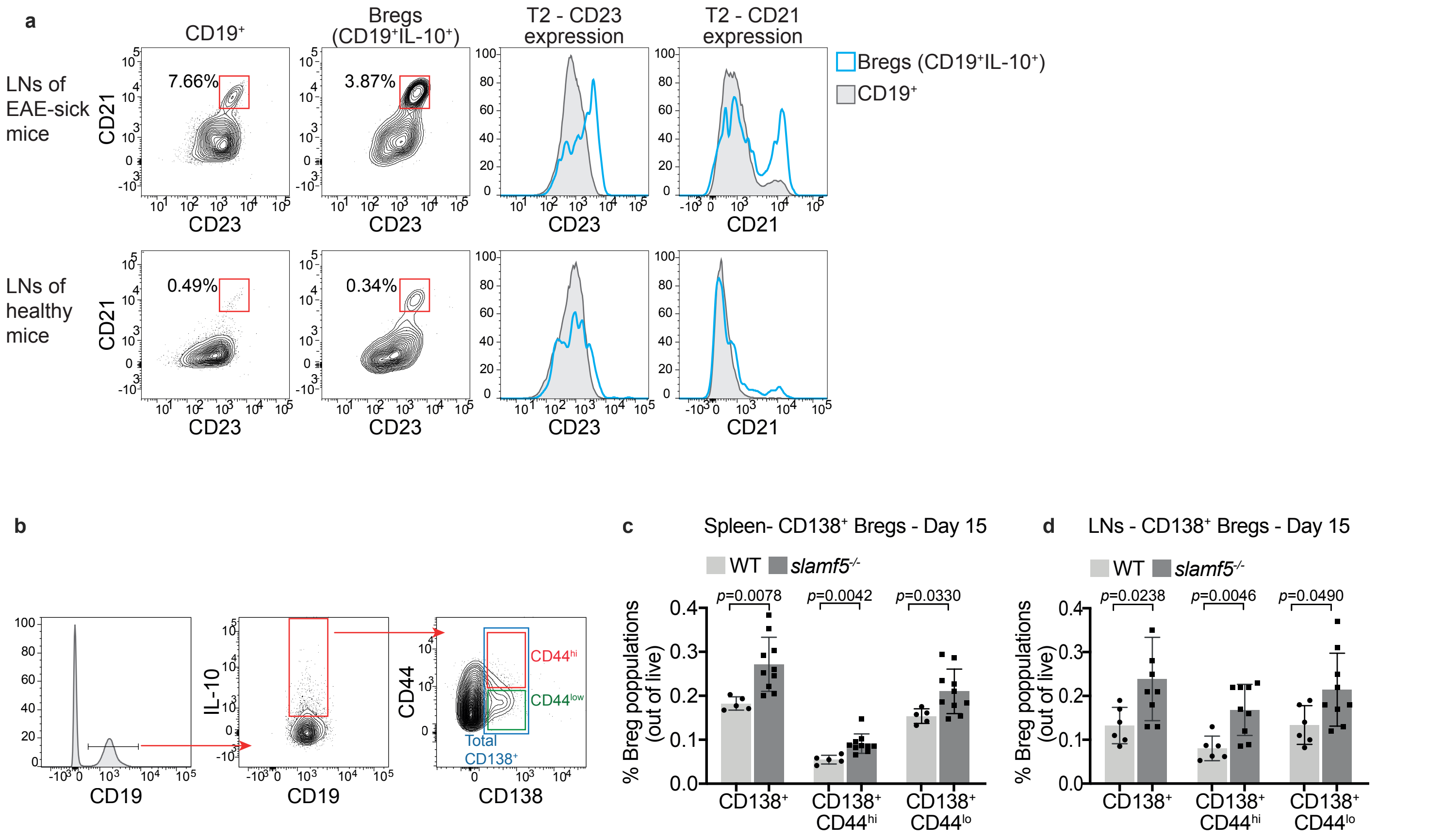

Supplementary Figure 3 – Breg subpopulations are increased in slamf5$^{-/-}$ mice during EAE

(**a**) WT mice induced with EAE were harvested at day 15. Lymph nodes (LNs) of the healthy and sick EAE mice were stained for the CD19$^+$CD21$^+$CD23$^+$IL-10$^+$ population. Representative CD23 CD21 dot plots were gated under CD19$^+$ or CD19$^+$IL-10$^+$ gate showing the percentage of CD23$^+$CD21$^+$ out of total CD19$^+$. Representative histograms of CD23 and CD21 in CD19$^+$ or CD19$^+$IL-10$^+$ populations are also shown. (**b-d**) WT and slamf5$^{-/-}$ mice were induced for EAE. On day 15, SLAMF5 expression was analysed on CD19$^+$CD138$^+$IL-10$^+$CD44^{hi} and CD19$^+$CD138$^+$IL-10$^+$CD44^{lo} populations. (**b**) showing gating strategy, (**c**) spleen analysis (WT n=5; slamf5$^{-/-}$ n=10 and (**d**) LNs analysis (WT n=6; slamf5$^{-/-}$ n=9, one experiment). Each dot represents a biological repeat. Data expressed as mean ± s.d (b-c). Unpaired Student's t-test

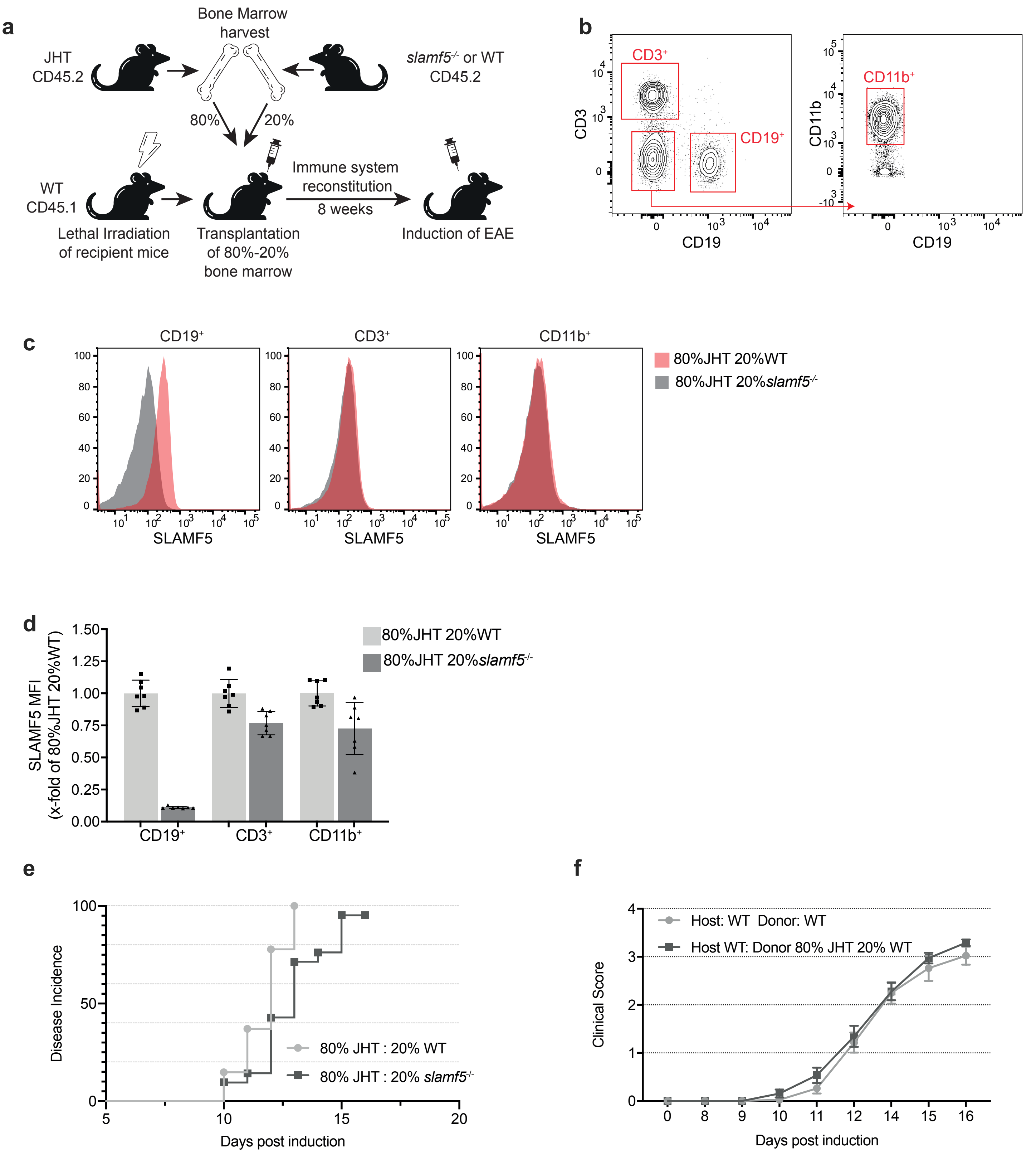

Supplementary Figure 4 – JHT Bone Marrow Chimera

CD45.1 WT were lethally irradiated and injected with BM consisting of 80% JHT BM and 20% WT CD45.2 or slamf5-/- BM. Following BM reconstitution, EAE was induced and followed for 15 days. (**a**) Diagram illustrating the experimental protocol. (**b-d**) SLAMF5 expression on CD19, CD3 and CD11b populations. (**b**) Gating strategy for CD19, CD3 and CD11b. (**c**) Representative histograms and (**d**) bar chart showing SLAMF5 MFI (20% WT n=7; 20% slamf5-/- n=7, one experiment); each dot represents a biological repeat. (e) Graph depicting the disease incidence of WT or slamf5-/- mice. Mice were considered sick with score >= 1 (20% WT n=27; 20% slamf5-/- n=21, three independent experiments). (**f**) Graph depicting the daily mean clinical score of two groups of EAE-induced chimeric mice: CD45.1 WT hosts transplanted with WT CD45.2 BM (n=20), and WT CD45.1 mice hosts transplanted with 80% JHT BM and 20%WT CD45.2 (n=29). Data expressed as mean ± s.d (d) or mean ± s.e.m (f).

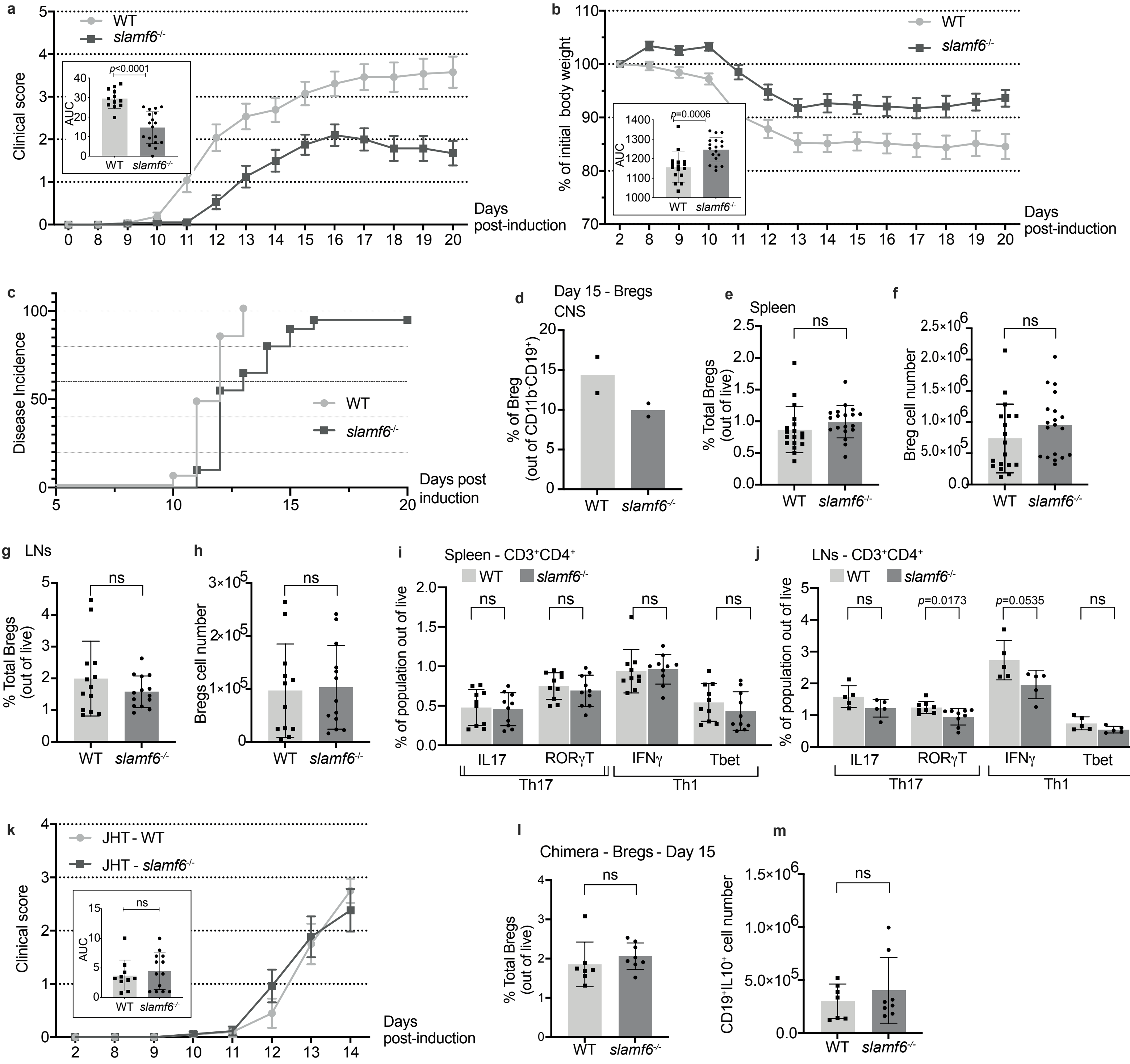

Supplementary Figure 5 - SLAMF6 protects mice from EAE but does not regulate Bregs

EAE was induced in WT and slamf6$^{-/-}$ mice. Mice were followed for 20 days. (**a**) Daily mean clinical scoring of the disease (WT n=12; slamf6$^{-/-}$ n=18, three independent experiments). (**b**) Weight loss shown as percent of initial body weight. (WT n=16; slamf6$^{-/-}$ n=18, three independent experiments). Insets depict the area under curve for days 0–20 (**c**) Graph depicting disease incidence. (WT n=12; slamf6$^{-/-}$ n=22, three independent experiments). (**d**) On day 15, the spinal cords were analyzed for the Breg population according the following markers: CD45$^+$CD11b$^-$CD19$^+$IL-10$^+$. Each dot represents a pool of 4-5 mice with similar disease score (n=2). (**e-h**) On day 15, spleens and LNs were analyzed. Total Breg (CD19$^+$IL-10$^+$) percentages and cell numbers in the (**e-f**) spleen (WT n=18; slamf6$^{-/-}$ n=19, three independent experiments) and (**g-h**) LNs (percentages: WT n=13; slamf6$^{-/-}$ n=14, count: WT n=12; slamf6$^{-/-}$ n=13, three independent experiments). (**i-j**) On day 15, spleens and LNs were collected, leukocytes were incubated overnight with MOG$_{35-55}$ and analyzed by flow cytometry for Th1 T cells (IL-17$^+$ and RORγT$^+$) and Th17 T cells (IFNγ$^+$ and T-bet$^+$); (**i**) spleen (WT n=10; slamf6$^{-/-}$ n=10, two independent experiments) and (**j**) LNs (WT n=5; slamf6$^{-/-}$ n=5; RORgT: WT n=8; slamf6$^{-/-}$ n=9, two independent experiments). (**k-l**) CD45.1 WT mice were lethally irradiated and injected with BM consisting of 80% JHT BM and 20% WT CD45.2 or slamf6$^{-/-}$ BM. Following BM reconstitution, EAE was induced, and mice were followed for 14 days. (**k**) Mean clinical scoring of the disease. Inset depicts the area under curve for days 0–14 (20% WT n= 10; 20% slamf6$^{-/-}$ n=13, two independent experiments). On day 15, Bregs were analyzed in the spleen by flow cytometry for (**l**) percentages and (**m**) numbers (20% WT n= 7; 20% slamf6$^{-/-}$ n=8, one experiments). Data expressed as mean ± s.d (e-j, k inset, m) or mean ± s.e.m (a,b,k). Unpaired Student's t-test with 95% confidence levels two-tailed (e-j, m). Mann–Whitney test (a,b,k insets).

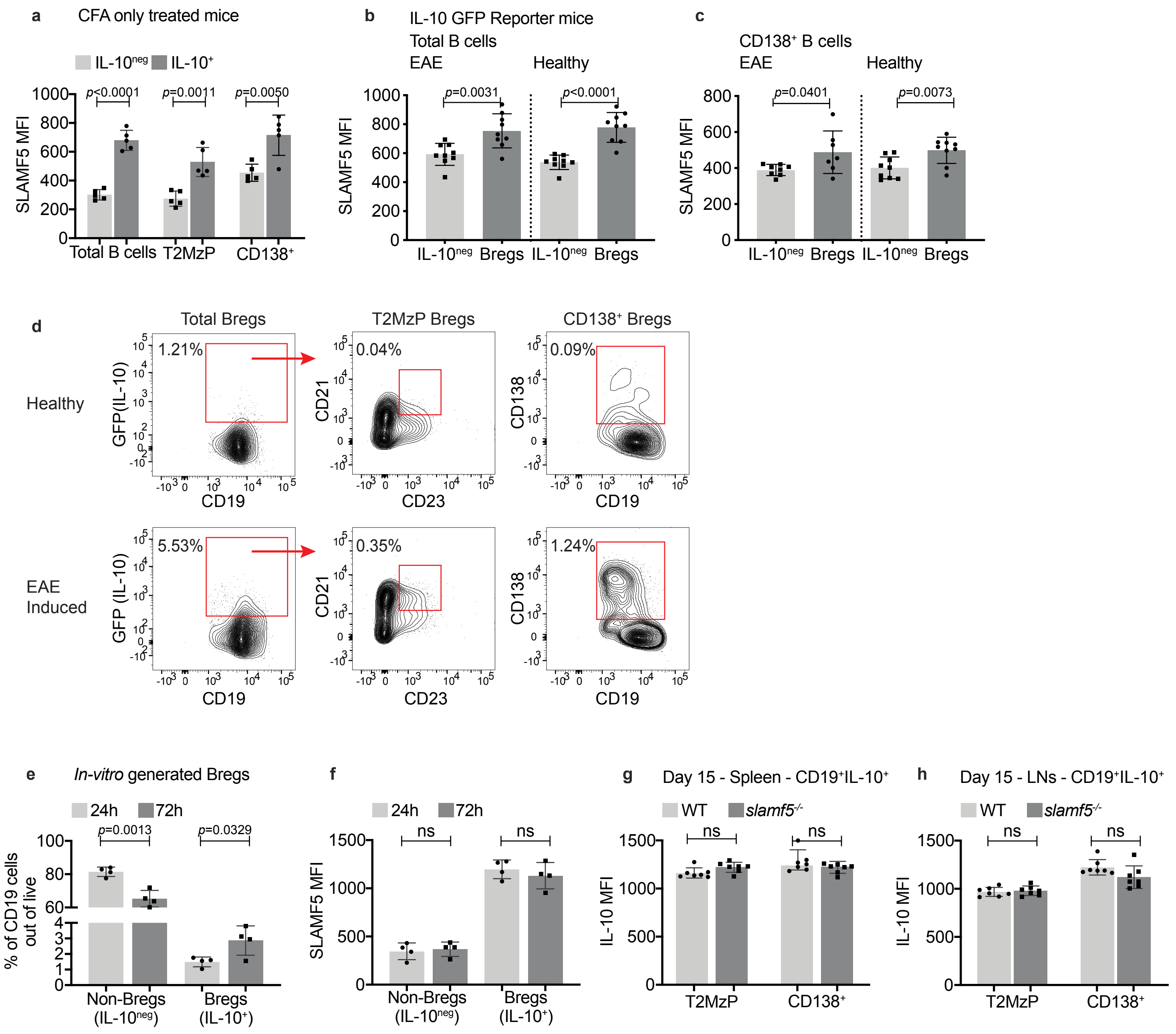

Supplementary Figure 6 - TLR4 activation does not regulate SLAMF5 expression on Bregs

(**a**) Mice were treated only with CFA. After 15 days, spleens were harvested and analyzed for SLAMF5 expression on B cells (cd19^{+}), T2MzP (CD19^{+}CD21^{+}CD23^{+}) and CD138^{+} B cells (CD19^{+}CD138^{+}) with comparison between non-Bregs (IL-10^{neg}) and Bregs (IL-10^{+}) (n=5, one experiment). Gating strategy is shown in Supplementary Figure 2b. (**b-d**) Spleens of Vert-x mice, healthy or EAE-induced, were harvested and analyzed for SLAMF5 expression on IL-10^{+} or IL-10^{neg} B cells. (**b**) Total B cells (CD19^{+}, n=9, two independent experiments) and (**c**) CD138^{+} B cells (CD19^{+}CD138^{+}, EAE IL10^{+} n=7; IL10^{neg} n=8; healthy n=9, two independent experiments). (d) Representative gating of Total Bregs (CD19^{+}GFP^{+}) and T2MzP Bregs (from the Total Bregs gate) and CD138^{+} Bregs (from the Total Bregs gate), showing percentages out of B cells, as discussed in Figure 3c-e. (**e-f**) Splenic B cells from naïve mice were purified and cultured with 10ug/ml LPS for 24 and 72 hrs, and with PMA, Ionomycin and Monensin for the last 5 hrs of the culture. The cells were analyzed by flow cytometry for (**e**) percentage of Breg (CD19^{+}IL-10^{+}) and non-Breg (CD19^{+}IL-10^{neg}) cells and for (**f**) SLAMF5 expression on Bregs and non-Bregs (n=4 , one experiment), gating strategy is shown in Supplementary Figure 8a. (**g-h**) WT and slamf5$^{-/-}$ mice were induced for EAE, at day 15. Mice were analyzed for IL-10 MFI in the T2MzP Breg and CD138^{+} Breg populations in the (**g**) spleen and (**h**) LNs (n=7, one experiment). Each dot represents one biological repeat, gating strategy for the populations is shown in Supplementary Figure 2b. Data expressed as mean ± s.d. Unpaired Student's t-test with 95% confidence levels two-tailed (a-c, e-h).

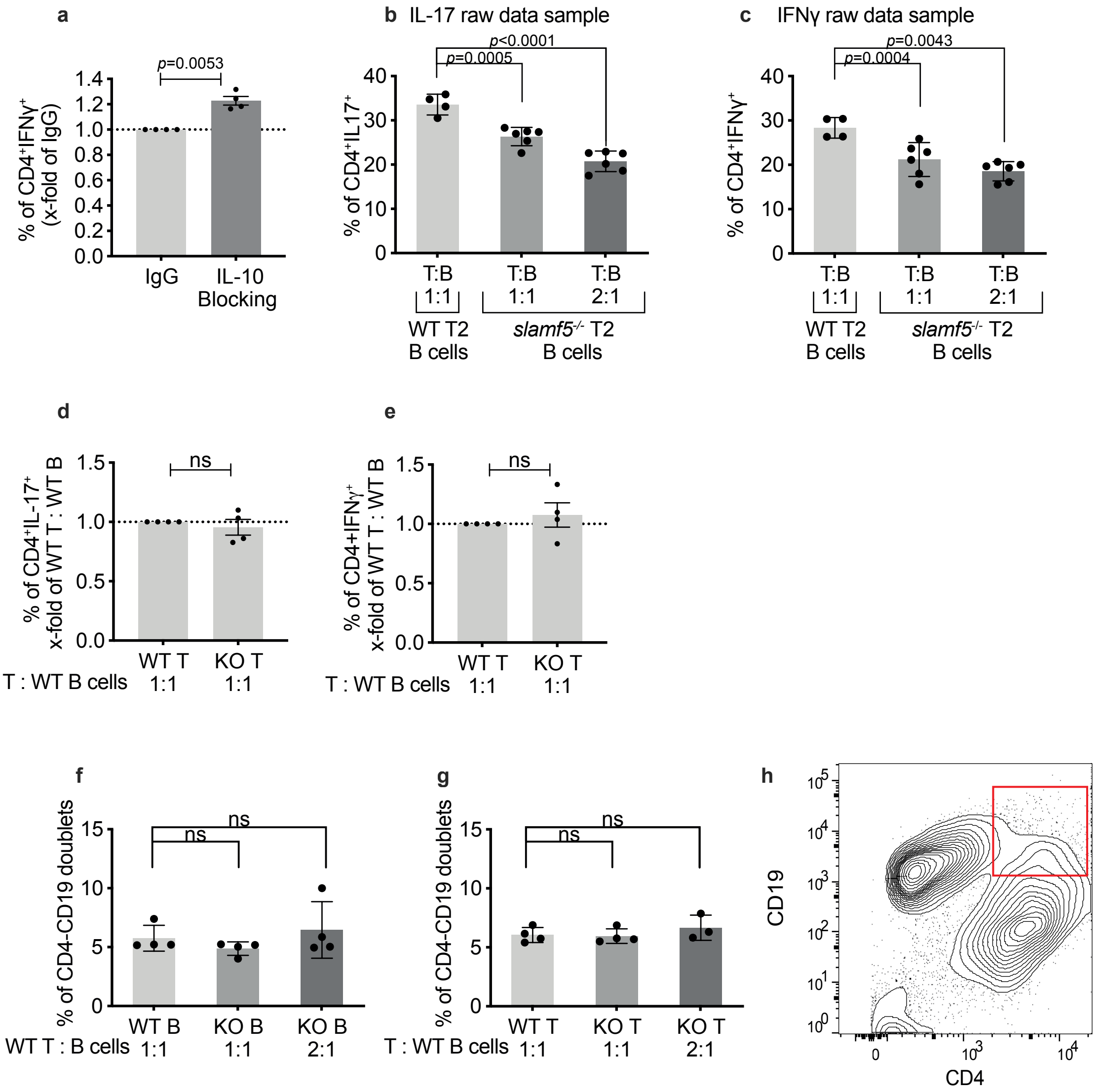

Supplementary Figure 7 - SLAMF5 deficient Bregs exhibit stronger suppressive capabilities

Sorted $CD4^+CD25^-$ splenic T cells from WT EAE-induced mice were co-cultured 1:1 or 2:1 for 72 hrs in the presence of anti-CD3 and anti-CD28 Abs with WT or $slamf5^{-/-}$ sorted splenic transitional 2 (T2) B cells ($CD19^+CD23^+CD21^+$). (**a**) WT T + WT T2 B cells in the suppression assay were incubated with 10ug/ml of IL-10 blocking Ab or IgG control. Graphs show percentages of $CD4^+$ IFNγ expression, x-fold of IgG (n=4, one experiment). (**b-c**) Sample of raw data for one individual experiment of Breg suppression (as shown in Figures 3 f-i) (**b**) IL-17 and (**c**) IFN-γ cytokine expression on $CD4^+$ T cells (WT T:WT B 1:1 n=4; WT T:$slamf5^{-/-}$ B 1:1 n=6; WT T:$slamf5^{-/-}$ B 2:1 n=6). (**d-e**) Sorted $CD4^+CD25^-$ splenic T cells of WT or $slamf5^{-/-}$ (indicated as “KO”) EAE-induced mice were co-cultured 1:1 for 72 hrs in the presence of anti-CD3 and anti-CD28 Abs with WT sorted splenic T2 B cells ($CD19^+CD23^+CD21^+$). Cells were analyzed for (**d**) IL-17 and (**e**) IFNγ expression shown as fold of the sample of “WT T and WT B” (n=4, one experiment). Gating strategy for a-e is shown in Supplementary Figure 1b. (**f-g**) After 72 hours, cells were analyzed for CD4-CD19 doublets, (**f**) WT T cells were cultured with WT or slamf5-/- T2 B cells (n=4, one experiment) or (**g**) $SLAMF5^{-/-}$ T cells were cultured with WT or $slamf5^{-/-}$ T2 B cells (WT T:WT B n=4; $slamf5^{-/-}$T:WT B 2:1 n=3, one experiment). (**h**) gating strategy for CD4-CD19 doublets shown in f-g. In the final 5 hrs, cells were cultured with PMA, Ionomycin, Monensin and Brefeldin A. Data expressed as mean ± s.d. Ratio paired t-test with 95% confidence levels two-tailed (a). Ordinary one-way ANOVA with Dunnett multiple comparison test (b,c,f,g) Unpaired Student’s t-test with 95% confidence levels two-tailed (d,e).

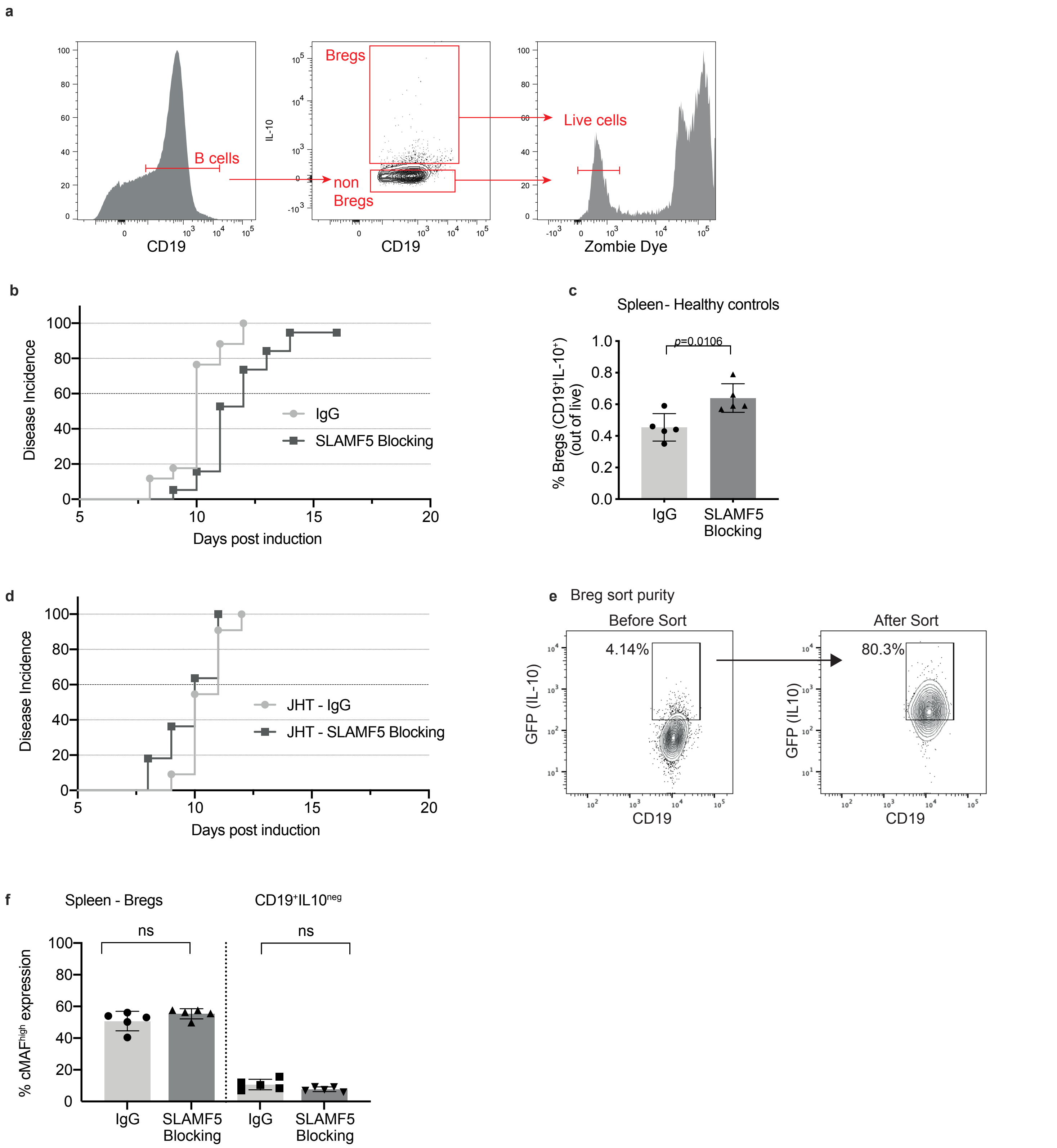

Supplementary Figure 8 – SLAMF5 Blocking

(**a**) Splenic B cells derived from EAE induced WT mice were cultured and treated with LPS and either SLAMF5 blocking or IgG control antibodies for 48 hours, in the presence of PMA, Ionomycin and Monensin for the final 5 hours. Cells were collected, and the survival of Bregs and non-Breg B cells ($CD19^{+}IL\text{-}10^{neg}$) was analyzed using Zombie dye by flow cytometry. Cells were gated as shown in Figures 4 a-f and Figure 6c,f. (**b**) EAE was induced in WT mice. On days 7, 9, and 11, the mice were injected i.v. with 30ug SLAMF5 blocking Ab or IgG. Graph depicting the disease incidence. Mice were considered sick with score >= 1 (IgG n=17; SLAMF5 blocking n=19, three independent experiments). (**c**) Control naïve healthy mice were treated with 30ug SLAMF5 blocking or IgG control Ab on the same injection schedule as the EAE-induced mice. Spleens were harvested after 15 days and analyzed for Breg ($CD19^{+}IL\text{-}10^{+}$) percentages. (IgG n=5; SLAMF5 blocking n=5, one experiment). (**d**) EAE was induced in JHT mice. On days 7, 9, and 11 the mice were injected i.v. with 30ug of SLAMF5 blocking Ab or IgG. Graph depicting disease incidence. Mice were considered sick with score >= 1. (IgG n=11; SLAMF5 blocking n=11, two independent experiments). (**e**) B cells pooled from two to three Vert-x mice in EAE remission were sorted for Bregs ($CD19^{+}GFP^{+}$), using the sorting strategy indicated. (**f**) Control naïve healthy mice were treated with 30ug SLAMF5 blocking and IgG on the same injection schedule as the EAE-induced mice. Spleens were harvested after 15 days and analyzed for c-Maf high expression in Bregs ($CD19^{+}IL\text{-}10^{+}$), and non-Bregs ($CD19^{+}IL\text{-}10^{neg}$) . (IgG n=5; SLAMF5 blocking n=5, one experiment). Data expressed as mean ± s.d. Unpaired Student's t-test with 95% confidence levels two-tailed (b,e).

a

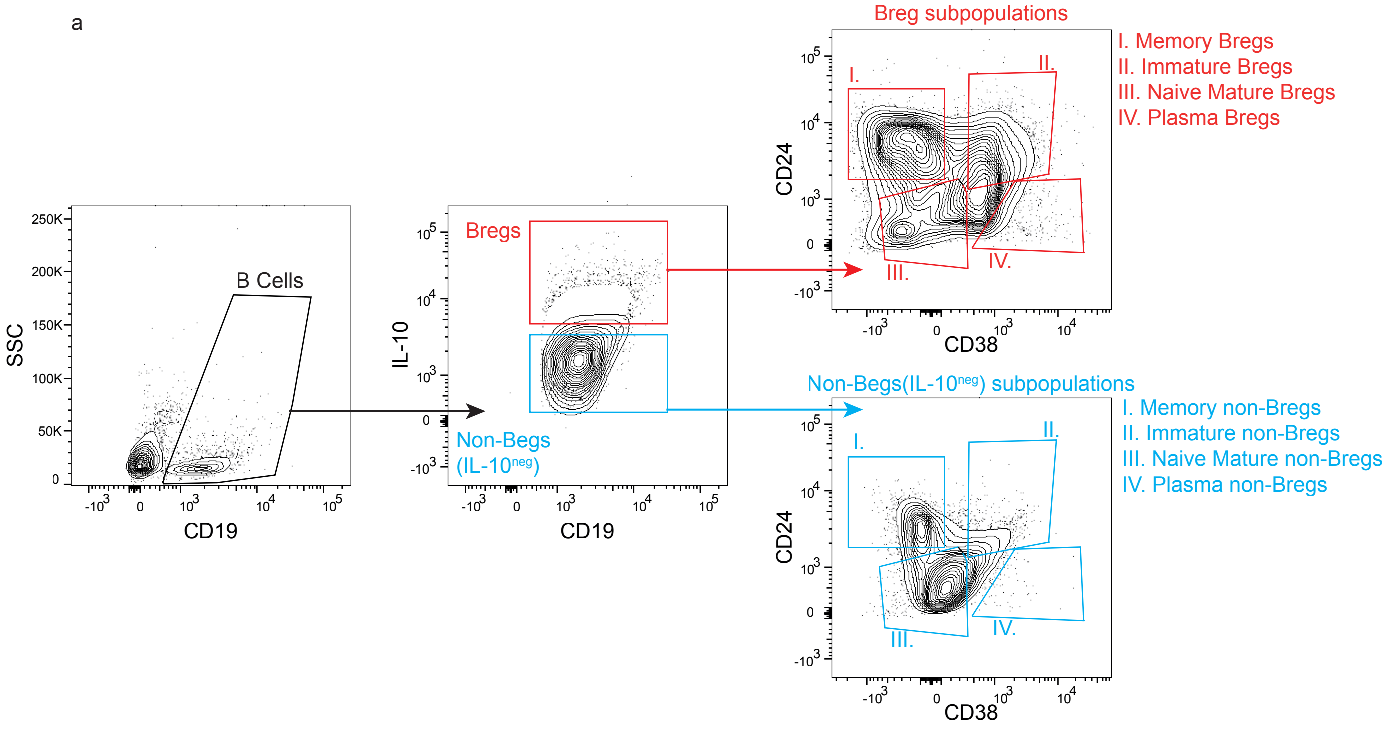

b

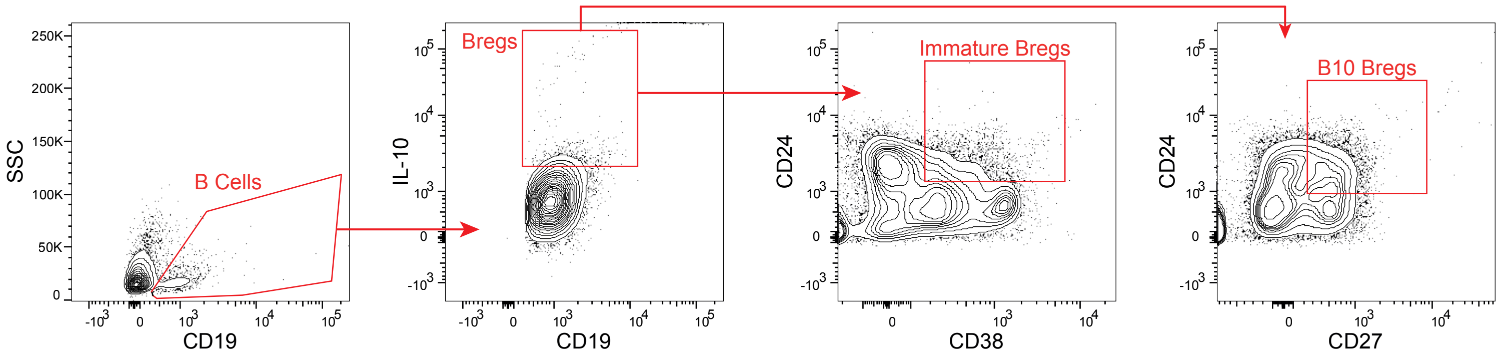

Supplementary Figure 9 – Human regulatory B cells gating

(**a**) Healthy human PBMCs were activated for 5 hours with PMA, Ionomycin, Brefeldin A and Monensin. Gating strategy used in Figure 6 a-b for Breg and non-Breg subpopulations; Total B cells: $CD19^{+}$; Memory B cells: $CD19^{+}CD24^{high}CD38^{low}$; naïve mature B cells: $CD19^{+}CD24^{low}CD38^{low}$; immature B cells: $CD19^{+}$ $CD24^{high}CD38^{high}$; and plasma B cells: $CD19^{+}CD24^{low}CD38^{high}$. Regulatory B cell subpopulations were analyzed with similar markers under the $CD19^{+}IL\text{-}10^{+}$ gate. (b) showing gating used in Figure 6 g-h of immature ($CD19^{+}IL\text{-}10^{+}CD24^{high}CD38^{high}$) and B10 ($CD19^{+}IL\text{-}10^{+}CD24^{high}CD27^{high}$) Breg populations .

Supplementary Table 1 – Patients list

Patient #	Sex	Age	Form of the Disease
P1	F	20	RR
P2	F	68	SP
P3	M	65	SP
P4	F	50	RR
P5	F	40	RR
P6	F	26	RR
P7	M	29	RR

PP: primary progressive MS patients.
SP: secondary progressive MS patients.
RR: relapsing-remitting MS patients.
M: male; F: female

Supplementary Table 2 – Antibodies List

	Antibody	fluorophore	clone	company
**Anti-mouse**	CD19	PE-CY7	ebio1d3	Invitrogen
	CD11b	APC-CY7	m1/70	Biolegend
	CD138	APC	281-2	BD-Biosciences
	CD138	PE	281-2	Biolegend
	CD21	APC-CY7	7e9	Biolegend
	CD23	FITC	b3b4	Biolegend
	CD24	Pacific Blue	m1/69	Biolegend
	CD25	APC	pc61.5	Invitrogen
	CD3	APC-CY7	145-2-c11	Biolegend
	CD4	APC	rm4-5	Biolegend
	CD4	FITC	rm4-5	Invitrogen
	CD45	FITC	ra3-6b2	Biolegend
	CPD	eFluor™ 450	-	Invitrogen
	FOXP3	PE	fjk-16s	Invitrogen
	Gata-3	APC	16e10a23	Biolegend
	IL10	Pacific Blue	jes5-16e3	Biolegend
	IL10	PE	jes5-16e3	BD-Biosciences
	Il17	PERCP-CY5.5	tc11-18h10.1	Biolegend
	IL4	PE-CY7	bvd6-24g2	Invitrogen
	INFγ	Pacific blue	xmg1.2	Biolegend
	MHCII	PE-CY7	AF6-120.1	Biolegend
	RORγt	BV421	q31-378	BD-Biosciences
	RORγt	PE	afkjs-9	Invitrogen
	SLAMF5	APC	REA212	Milteny
	T-bet	PE-CY7	ebio4b10	Invitrogen
	TNFα	PE	MP6-xt22	Biolegend
	Zombie violet		-	Biolegend
**Anti-human**	CD19	FITC	h1b-19	Biolegend
	CD38	BV421	hit2	Biolegend
	CD27	APC-CY7	o323	Biolegend
	CD24	PE-CY7	ml5	Biolegend
	SLAMF5	PE	MZ18-21F6	Milteny
	IL10	APC	JES3-19F1	BD-Biosciences
**Anti-mouse/human**	cMAF	unconjugated	Rabbit Polyclonal	Abcam
	Rabbit IgG		polyclonal	Biolegend

Supplementary Table 3 – qPCR primers

	Gene	Primer sequence	
**Mouse**	L32	F TTAAGCGAAACTGGCGGGAAAC	R-TTGTTGCTCCCATAACCGATG
	BCL-2	F GCTACCGTCGTGACTT	R GCCGGTTCAGGTACTC
	BCL-XL	F AACATCCCAGCTTCACATAACCC	R GCGACCCCAGTTTACTCCATCC
	cMAF	F AGGAGGTGATCCGACTGAAGCA	R TCTCCTGCTTGAGGTGGTCTAC
	AhR	F AGGATCGGGGTACCAGTTCA	R CTCCAGCGACTGTGTTTTGC
**Human**	L32	F ACAAAGCACATGCTGCCCAGTG	R TTCCACGATGGCTTTGCGGTTC
	cMAF	F AGAAGTTGGTGAGCAGCGGCTT	R CACTGATGGCTCCAACTTGCGA
