# Supplementary material for: The survival and function of IL-10-producing regulatory B cells are negatively controlled by SLAMF5
Source: Nat Commun. 2021 Mar 25;12:1893. doi: 10.1038/s41467-021-22230-z (PMC7994628; doi:10.1038/s41467-021-22230-z)
Supplement: Supplementary file 2 — Reporting Summary [file 41467_2021_22230_MOESM2_ESM.pdf]

nature research

Corresponding author(s): Idit Shachar

Last updated by author(s): Feb 22, 2021

Reporting Summary

Nature Research wishes to improve the reproducibility of the work that we publish. This form provides structure for consistency and transparency in reporting. For further information on Nature Research policies, see our Editorial Policies and the Editorial Policy Checklist.

Statistics

For all statistical analyses, confirm that the following items are present in the figure legend, table legend, main text, or Methods section.

n/a	Confirmed	
☐	☒	The exact sample size (*n*) for each experimental group/condition, given as a discrete number and unit of measurement
☐	☒	A statement on whether measurements were taken from distinct samples or whether the same sample was measured repeatedly
☐	☒	The statistical test(s) used AND whether they are one- or two-sided<br>*Only common tests should be described solely by name; describe more complex techniques in the Methods section.*
☒	☐	A description of all covariates tested
☒	☐	A description of any assumptions or corrections, such as tests of normality and adjustment for multiple comparisons
☐	☒	A full description of the statistical parameters including central tendency (e.g. means) or other basic estimates (e.g. regression coefficient) AND variation (e.g. standard deviation) or associated estimates of uncertainty (e.g. confidence intervals)
☐	☒	For null hypothesis testing, the test statistic (e.g. *F*, *t*, *r*) with confidence intervals, effect sizes, degrees of freedom and *P* value noted<br>*Give P values as exact values whenever suitable.*
☒	☐	For Bayesian analysis, information on the choice of priors and Markov chain Monte Carlo settings
☒	☐	For hierarchical and complex designs, identification of the appropriate level for tests and full reporting of outcomes
☒	☐	Estimates of effect sizes (e.g. Cohen's *d*, Pearson's *r*), indicating how they were calculated

Our web collection on statistics for biologists contains articles on many of the points above.

Software and code

Policy information about availability of computer code

Data collection	FACSDiva 8 was used for FACS data collection. LightCycler® 480 software was used for qPCR data collection. BD Canto used for FACS analysis and BD FACSMelody was used for sorting.
Data analysis	LightCycler® 480 software was usedqPCR analysis, Flowjo v10 was used for FACS analysis, Graphpad Prism v8 was used for statistical analysis. For RNAseq analysis the following were used: UTAP pipeline (the Weizmann Institute Bioinformatics Unit),cutadapt, STAR v2.4.2a, DESeq2, fdrtool

For manuscripts utilizing custom algorithms or software that are central to the research but not yet described in published literature, software must be made available to editors and reviewers. We strongly encourage code deposition in a community repository (e.g. GitHub). See the Nature Research guidelines for submitting code & software for further information.

Data

Policy information about availability of data

All manuscripts must include a data availability statement. This statement should provide the following information, where applicable:

- Accession codes, unique identifiers, or web links for publicly available datasets
- A list of figures that have associated raw data
- A description of any restrictions on data availability

RNA sequencing data discussed in this publication have been deposited in NCBI's Gene Expression Omnibus and are accessible through GEO series accession number GSE160828

Field-specific reporting

Please select the one below that is the best fit for your research. If you are not sure, read the appropriate sections before making your selection.

- [x] Life sciences
- [ ] Behavioural & social sciences
- [ ] Ecological, evolutionary & environmental sciences

For a reference copy of the document with all sections, see nature.com/documents/nr-reporting-summary-flat.pdf

Life sciences study design

All studies must disclose on these points even when the disclosure is negative.

Sample size	The group size used for all experiments ensured statistical significance of the results.<br>Each in vitro experiment was performed at least four times. Due the variability of EAE each of the in vivo experiments included a large cohort of mice.<br>For charcterization of Multiple sclerosis patients experiments, group size was limited by the availability of patient samples.<br>Sample size calculations were not performed
Data exclusions	In most experiments no data was excluded.<br>In few experiments outliers (3-fold or more from the mean of the group) were excluded.<br>Experiments were samples were excluded: Fig.4 a, a bilogical repeat was removed from the experiment (same donor from both treatment groups). Fig. 2J,two biological repeats were ecxluded.
Replication	To verify the reproducibility, experiments were performed three to five times. Only the RNAseq experiment was preformed twice.<br>Replication attempts were successfull
Randomization	Mice were randomly distributed amongst groups at the start of each experiment.<br>For in vitro assays, cells were randomly assigned to either experiment or control groups. A biological sample was split into equall samples for control and experiment groups.
Blinding	Blind scoring was done in the EAE mice model.

Behavioural & social sciences study design

All studies must disclose on these points even when the disclosure is negative.

Study description	*Briefly describe the study type including whether data are quantitative, qualitative, or mixed-methods (e.g. qualitative cross-sectional, quantitative experimental, mixed-methods case study).*
Research sample	*State the research sample (e.g. Harvard university undergraduates, villagers in rural India) and provide relevant demographic information (e.g. age, sex) and indicate whether the sample is representative. Provide a rationale for the study sample chosen. For studies involving existing datasets, please describe the dataset and source.*
Sampling strategy	*Describe the sampling procedure (e.g. random, snowball, stratified, convenience). Describe the statistical methods that were used to predetermine sample size OR if no sample-size calculation was performed, describe how sample sizes were chosen and provide a rationale for why these sample sizes are sufficient. For qualitative data, please indicate whether data saturation was considered, and what criteria were used to decide that no further sampling was needed.*
Data collection	*Provide details about the data collection procedure, including the instruments or devices used to record the data (e.g. pen and paper, computer, eye tracker, video or audio equipment) whether anyone was present besides the participant(s) and the researcher, and whether the researcher was blind to experimental condition and/or the study hypothesis during data collection.*
Timing	*Indicate the start and stop dates of data collection. If there is a gap between collection periods, state the dates for each sample cohort.*
Data exclusions	*If no data were excluded from the analyses, state so OR if data were excluded, provide the exact number of exclusions and the rationale behind them, indicating whether exclusion criteria were pre-established.*
Non-participation	*State how many participants dropped out/declined participation and the reason(s) given OR provide response rate OR state that no participants dropped out/declined participation.*
Randomization	*If participants were not allocated into experimental groups, state so OR describe how participants were allocated to groups, and if allocation was not random, describe how covariates were controlled.*

Ecological, evolutionary & environmental sciences study design

All studies must disclose on these points even when the disclosure is negative.

Study description	*Briefly describe the study. For quantitative data include treatment factors and interactions, design structure (e.g. factorial, nested, hierarchical), nature and number of experimental units and replicates.*
Research sample	*Describe the research sample (e.g. a group of tagged Passer domesticus, all Stenocereus thurberi within Organ Pipe Cactus National Monument), and provide a rationale for the sample choice. When relevant, describe the organism taxa, source, sex, age range and any manipulations. State what population the sample is meant to represent when applicable. For studies involving existing datasets, describe the data and its source.*
Sampling strategy	*Note the sampling procedure. Describe the statistical methods that were used to predetermine sample size OR if no sample-size calculation was performed, describe how sample sizes were chosen and provide a rationale for why these sample sizes are sufficient.*
Data collection	*Describe the data collection procedure, including who recorded the data and how.*
Timing and spatial scale	*Indicate the start and stop dates of data collection, noting the frequency and periodicity of sampling and providing a rationale for these choices. If there is a gap between collection periods, state the dates for each sample cohort. Specify the spatial scale from which the data are taken*
Data exclusions	*If no data were excluded from the analyses, state so OR if data were excluded, describe the exclusions and the rationale behind them, indicating whether exclusion criteria were pre-established.*
Reproducibility	*Describe the measures taken to verify the reproducibility of experimental findings. For each experiment, note whether any attempts to repeat the experiment failed OR state that all attempts to repeat the experiment were successful.*
Randomization	*Describe how samples/organisms/participants were allocated into groups. If allocation was not random, describe how covariates were controlled. If this is not relevant to your study, explain why.*
Blinding	*Describe the extent of blinding used during data acquisition and analysis. If blinding was not possible, describe why OR explain why blinding was not relevant to your study.*

Did the study involve field work? ☐ Yes ☐ No

Field work, collection and transport

Field conditions	*Describe the study conditions for field work, providing relevant parameters (e.g. temperature, rainfall).*
Location	*State the location of the sampling or experiment, providing relevant parameters (e.g. latitude and longitude, elevation, water depth).*
Access & import/export	*Describe the efforts you have made to access habitats and to collect and import/export your samples in a responsible manner and in compliance with local, national and international laws, noting any permits that were obtained (give the name of the issuing authority, the date of issue, and any identifying information).*
Disturbance	*Describe any disturbance caused by the study and how it was minimized.*

Reporting for specific materials, systems and methods

We require information from authors about some types of materials, experimental systems and methods used in many studies. Here, indicate whether each material, system or method listed is relevant to your study. If you are not sure if a list item applies to your research, read the appropriate section before selecting a response.

Materials & experimental systems

n/a	Involved in the study	
☐	☒	Antibodies
☒	☐	Eukaryotic cell lines
☒	☐	Palaeontology and archaeology
☐	☒	Animals and other organisms
☐	☒	Human research participants
☒	☐	Clinical data
☒	☐	Dual use research of concern

Methods

n/a	Involved in the study	
☒	☐	ChIP-seq
☐	☒	Flow cytometry
☒	☐	MRI-based neuroimaging

Antibodies

Antibodies used

Anti mouse FACS antibodies:
Antigen fluorophore clone company catalog lot
CD19 PE-CY7 ebio1d3 Invitrogen 25-0193-82 4341605
CD11b APC-CY7 m1/70 Biolegend 101226 B273857
CD138 APC 281-2 BD-Biosciences 558626 7096640
CD138 PE 281-2 Biolegend 142504 B246403
CD21 APC-CY7 7e9 Biolegend 123418 B251995
CD23 FITC b3b4 Biolegend 101606 B218393
CD24 Pacific Blue m1/69 Biolegend 101820 B271348
CD25 APC pc61.5 Invitrogen 17-0251-81A 4329878
CD3 APC-CY7 145-2-c11 Biolegend 100330 B265548
CD4 APC rm4-5 Biolegend 100516 B218256
CD4 FITC rm4-5 Invitrogen 11-0042-81A 4276358
FOXP3 PE fjk-16s Invitrogen 12-5773-80A 4307349
Gata-3 APC 16e10a23 Biolegend 653806 B229654
IL10 Pacific Blue jes5-16e3 Biolegend 505020 B284984
IL10 PE jes5-16e3 BD-Biosciences 561060 7136549
Il17 PERCP-CY5.5 tc11-18h10.1 Biolegend 506920 B257639
IL4 PE-CY7 bvd6-24g2 Invitrogen 25-7042-82 E07663-1636
INFγ Pacific blue xmg1.2 Biolegend 505818 B234191
MHCII PE-CY7 AF6-120.1 Biolegend 116420 B249894
RORγt BV421 q31-378 BD-Biosciences 562894 7045532
RORγt PE afkjs-9 Invitrogen 12-6988-82 4274957
SLAMF5 APC REA212 Milteny 130-102-592 5190207224
T-bet PE-CY7 ebio4b10 Invitrogen 25-5825-82 4341622
TNFα PE MP6-xt22 Biolegend 506306 B183524
Zombie violet - Biolegend 77477 B274542

Anti human FACS antibodies:
Antigen fluorophore clone company catalog lot
CD19 FITC h1b-19 Biolegend 302206 B223041
CD38 BV421 hit2 Biolegend 303526 B240476
CD27 APC-CY7 o323 Biolegend 302816 B169307
CD24 PE-CY7 ml5 Biolegend 311120 B242172
SLAMF5 PE MZ18-21F6 Milteny 130-093-277 5190131145
IL10 APC JES3-19F1 BD-Biosciences 554707 7124679

T cell activation for Regulatory B cell suppression:
Anti CD3 145-2c11 biolegend 100340 B260927

SLAMF5 Blocking:
IgG2a MG2a-53 biolegend 401509 B304914
Anti-SLAMF5 (B4) B4 crownbio B4-migg2a AB190038/39

Validation

All used antibodies were validated commercially. For SLAMF5 blocking antibody validation: https://doi.org/10.1038/onc.2013.31

Eukaryotic cell lines

Policy information about cell lines

Cell line source(s)

State the source of each cell line used.

Authentication

Describe the authentication procedures for each cell line used OR declare that none of the cell lines used were authenticated.

Mycoplasma contamination

Confirm that all cell lines tested negative for mycoplasma contamination OR describe the results of the testing for mycoplasma contamination OR declare that the cell lines were not tested for mycoplasma contamination.

Commonly misidentified lines
(See ICLAC register)

Name any commonly misidentified cell lines used in the study and provide a rationale for their use.

Palaeontology and Archaeology

Specimen provenance	*Provide provenance information for specimens and describe permits that were obtained for the work (including the name of the issuing authority, the date of issue, and any identifying information).*
Specimen deposition	*Indicate where the specimens have been deposited to permit free access by other researchers.*
Dating methods	*If new dates are provided, describe how they were obtained (e.g. collection, storage, sample pretreatment and measurement), where they were obtained (i.e. lab name), the calibration program and the protocol for quality assurance OR state that no new dates are provided.*

☐ Tick this box to confirm that the raw and calibrated dates are available in the paper or in Supplementary Information.

Ethics oversight	*Identify the organization(s) that approved or provided guidance on the study protocol, OR state that no ethical approval or guidance was required and explain why not.*

Note that full information on the approval of the study protocol must also be provided in the manuscript.

Animals and other organisms

Policy information about studies involving animals; ARRIVE guidelines recommended for reporting animal research

Laboratory animals	Mice used in experiments: C57BL/6 WT, C57BL/6 CD45.1, SLAMF5-/-, JHT, Vertex, all: Males or Females, 6-12 weeks.<br>All mice were sex and age matched.
Wild animals	No wild animals were involved
Field-collected samples	This study did not involve field collected samples
Ethics oversight	All animal procedures were approved by the Animal Research Committee at the Weizmann Institute of Science

Note that full information on the approval of the study protocol must also be provided in the manuscript.

Human research participants

Policy information about studies involving human research participants

Population characteristics	Healthy donor samples - The study was performed on blood cells from healthy donors. For most healthy donors buffy coats were provided by the Blood Bank (MDA Israel Magen David Adom), no additional information (age, sex) was given for these donors. These samples were recived without details from the blood bank<br><br>For Multiple Sclerosis patients: peripheal blood charcterization. Blood samples of newly diagnosed Multiple Sclerosis patients and matched (age/sex) controls were recieved from the department of Neurology, Barzilai University Medical Center, Israel.<br><br>Multiple Sclerosis samples:samples were collected from newly diagnosed patients that were not treated yet. Normal controls were sec/age matched accordingly.
Recruitment	Buffy coats for blood cell isolation from healthy blood donors were provided by the Blood Bank (MDA Israel Magen David Adom)<br>Blood samples of newly diagnosed and yet to be treated Multiple Sclerosis patients and matched (age/sex) controls were recived.<br>Recruitment occurred when patients came to the Centre for Rheumatology for their first treatment, and blood samples were taken before the treatment.
Ethics oversight	Institutional Review Board of the Barzilai Medical Center, Ashkelon, Israel.

Note that full information on the approval of the study protocol must also be provided in the manuscript.

Clinical data

Policy information about clinical studies

All manuscripts should comply with the ICMJEguidelines for publication of clinical research and a completedCONSORT checklist must be included with all submissions.

Clinical trial registration	*Provide the trial registration number from ClinicalTrials.gov or an equivalent agency.*
Study protocol	*Note where the full trial protocol can be accessed OR if not available, explain why.*
Data collection	*Describe the settings and locales of data collection, noting the time periods of recruitment and data collection.*
Outcomes	*Describe how you pre-defined primary and secondary outcome measures and how you assessed these measures.*

Dual use research of concern

Policy information about dual use research of concern

Hazards

Could the accidental, deliberate or reckless misuse of agents or technologies generated in the work, or the application of information presented in the manuscript, pose a threat to:

No	Yes	
☐	☐	Public health
☐	☐	National security
☐	☐	Crops and/or livestock
☐	☐	Ecosystems
☐	☐	Any other significant area

Experiments of concern

Does the work involve any of these experiments of concern:

No	Yes	
☐	☐	Demonstrate how to render a vaccine ineffective
☐	☐	Confer resistance to therapeutically useful antibiotics or antiviral agents
☐	☐	Enhance the virulence of a pathogen or render a nonpathogen virulent
☐	☐	Increase transmissibility of a pathogen
☐	☐	Alter the host range of a pathogen
☐	☐	Enable evasion of diagnostic/detection modalities
☐	☐	Enable the weaponization of a biological agent or toxin
☐	☐	Any other potentially harmful combination of experiments and agents

ChIP-seq

Data deposition

☐ Confirm that both raw and final processed data have been deposited in a public database such as GEO.

☐ Confirm that you have deposited or provided access to graph files (e.g. BED files) for the called peaks.

Data access links
May remain private before publication.
For "Initial submission" or "Revised version" documents, provide reviewer access links. For your "Final submission" document, provide a link to the deposited data.

Files in database submission
Provide a list of all files available in the database submission.

Genome browser session
(e.g. UCSC)
Provide a link to an anonymized genome browser session for "Initial submission" and "Revised version" documents only, to enable peer review. Write "no longer applicable" for "Final submission" documents.

Methodology

Replicates
Describe the experimental replicates, specifying number, type and replicate agreement.

Sequencing depth
Describe the sequencing depth for each experiment, providing the total number of reads, uniquely mapped reads, length of reads and whether they were paired- or single-end.

Antibodies
Describe the antibodies used for the ChIP-seq experiments; as applicable, provide supplier name, catalog number, clone name, and lot number.

Peak calling parameters
Specify the command line program and parameters used for read mapping and peak calling, including the ChIP, control and index files used.

Data quality
Describe the methods used to ensure data quality in full detail, including how many peaks are at FDR 5% and above 5-fold enrichment.

Software
Describe the software used to collect and analyze the ChIP-seq data. For custom code that has been deposited into a community repository, provide accession details.

Flow Cytometry

Plots

Confirm that:

- [ ] The axis labels state the marker and fluorochrome used (e.g. CD4-FITC).
- [x] The axis scales are clearly visible. Include numbers along axes only for bottom left plot of group (a 'group' is an analysis of identical markers).
- [x] All plots are contour plots with outliers or pseudocolor plots.
- [x] A numerical value for number of cells or percentage (with statistics) is provided.

Methodology

Sample preparation

Murine Cell Preparation - Murine spleens and draining lymph nodes were dissected and collected in PBS (Biological Industries) with 2% FCS. Organs were processed through a 100-µm-cell strainer, and treated with Red Blood Lysis buffer to lyse erythrocytes. B cells were then purified by positive B cell selection with B220 magnetic beads (BD Biosciences). Spinal Cords (SC) were dissected into RPMI, by flushing the spinal cord out of the spine with RPMI (Gibco) using an 18-gauge needle. SCs were processed in a tissue grinder and transferred to a 30 ml tube containing 20 ml RPMI 1640, 9 ml Percoll (Sigma-Aldrich) and 1 ml 10X PBS. Cells were then centrifuged at 7800g for 30 min at RT. The middle fraction was collected, washed twice with PBS 2% FCS before underlaying with 1 ml Ficoll-Paque Plus (GE Healthcare). Cells were then centrifuged at 1400g for 25 min at RT with no brake. The middle interface, containing leukocytes was collected into tubes. Due to the low lymphocyte yield from CNS tissue, 4-5 spinal cords were combined for each measurement.

Human cell preparation - sampels were centrifuged at 2500RPM 5 min in order to better separate the buffy coat, which was then collec ted, washed and treated with BD Pharm Lyse™ (BD-Bioscience) to lyse red blood cells.

Instrument

FACS Canto (BD Biosciences)

Software

FACSDiva v.8 for data collection, FlowJo™ v.10 for data abalysis.

Cell population abundance

Sorted GFP+B cells from Vertex mice (IL-10+ B cells) were aprox. 4% of the pre-sorted population (enriched B220+ subsets by b220 MACS beads, BDbiosciences) and were aprox. 80% pure post sort.

Gating strategy

For all samples the following gating strategy was used, exclusion of doublets (FSC-H/FSC-A), lymphocytes(SSC-A/FSC-A).
Spleen/LN T cell populations: ->double positive for CD3+/CD4+ in an area plot -> area plot of CD4+ and target marker, poistive gating for marker was done according to controls.
Spinal cord T cell population shown in Supp. Fig 1.
Spleen/LN Regulatory B cells: Supp. Fig 2.
Spinal cord Regulatory B cell shown in Supp. Fig. 1
Human PB Regulatory B cells: -> SSC-A/CD19+ -> various B cell populations as described in the legend of Fig. 6 -> CD19+IL10+ in area plot compared to control.

- [x] Tick this box to confirm that a figure exemplifying the gating strategy is provided in the Supplementary Information.

Magnetic resonance imaging

Experimental design

Design type

Indicate task or resting state; event-related or block design.

Design specifications

Specify the number of blocks, trials or experimental units per session and/or subject, and specify the length of each trial or block (if trials are blocked) and interval between trials.

Behavioral performance measures

State number and/or type of variables recorded (e.g. correct button press, response time) and what statistics were used to establish that the subjects were performing the task as expected (e.g. mean, range, and/or standard deviation across subjects).

Acquisition

Imaging type(s) | *Specify: functional, structural, diffusion, perfusion.*

Field strength | *Specify in Tesla*

Sequence & imaging parameters | *Specify the pulse sequence type (gradient echo, spin echo, etc.), imaging type (EPI, spiral, etc.), field of view, matrix size, slice thickness, orientation and TE/TR/flip angle.*

Area of acquisition | *State whether a whole brain scan was used OR define the area of acquisition, describing how the region was determined.*

Diffusion MRI ☐ Used ☐ Not used

Preprocessing

Preprocessing software | *Provide detail on software version and revision number and on specific parameters (model/functions, brain extraction, segmentation, smoothing kernel size, etc.).*

Normalization | *If data were normalized/standardized, describe the approach(es): specify linear or non-linear and define image types used for transformation OR indicate that data were not normalized and explain rationale for lack of normalization.*

Normalization template | *Describe the template used for normalization/transformation, specifying subject space or group standardized space (e.g. original Talairach, MNI305, ICBM152) OR indicate that the data were not normalized.*

Noise and artifact removal | *Describe your procedure(s) for artifact and structured noise removal, specifying motion parameters, tissue signals and physiological signals (heart rate, respiration).*

Volume censoring | *Define your software and/or method and criteria for volume censoring, and state the extent of such censoring.*

Statistical modeling & inference

Model type and settings | *Specify type (mass univariate, multivariate, RSA, predictive, etc.) and describe essential details of the model at the first and second levels (e.g. fixed, random or mixed effects; drift or auto-correlation).*

Effect(s) tested | *Define precise effect in terms of the task or stimulus conditions instead of psychological concepts and indicate whether ANOVA or factorial designs were used.*

Specify type of analysis: ☐ Whole brain ☐ ROI-based ☐ Both

Statistic type for inference (See Eklund et al. 2016) | *Specify voxel-wise or cluster-wise and report all relevant parameters for cluster-wise methods.*

Correction | *Describe the type of correction and how it is obtained for multiple comparisons (e.g. FWE, FDR, permutation or Monte Carlo).*

Models & analysis

n/a	Involved in the study
☐	☐ Functional and/or effective connectivity
☐	☐ Graph analysis
☐	☐ Multivariate modeling or predictive analysis

Functional and/or effective connectivity | *Report the measures of dependence used and the model details (e.g. Pearson correlation, partial correlation, mutual information).*

Graph analysis | *Report the dependent variable and connectivity measure, specifying weighted graph or binarized graph, subject- or group-level, and the global and/or node summaries used (e.g. clustering coefficient, efficiency, etc.).*

Multivariate modeling and predictive analysis | *Specify independent variables, features extraction and dimension reduction, model, training and evaluation metrics.*
